# Anionic Surfactant‐Modulated Electrode–Electrolyte Interface Promotes H_2_O_2_ Electrosynthesis

**DOI:** 10.1002/advs.202405474

**Published:** 2024-07-25

**Authors:** Wen Sun, Lei Tang, Wangxin Ge, Yu Fan, Xuedi Sheng, Lei Dong, Wenfei Zhang, Hongliang Jiang, Chunzhong Li

**Affiliations:** ^1^ Key Laboratory for Ultrafine Materials of Ministry of Education School of Chemical Engineering East China University of Science and Technology Shanghai 200237 China; ^2^ Shanghai Engineering Research Center of Hierarchical Nanomaterials School of Materials Science and Engineering East China University of Science and Technology Shanghai 200237 China

**Keywords:** electrode–electrolyte interface, electrolyte engineering, H_2_O_2_ electrosynthesis, in situ spectroscopy, solvation structure

## Abstract

Conventional strategies for highly selective and active hydrogen peroxide (H_2_O_2_) electrosynthesis primarily focus on catalyst design. Electrocatalytic reactions take place at the electrified electrode–electrolyte interface. Well‐designed electrolytes, when combined with commercial catalysts, can be directly applied to high‐efficiency H_2_O_2_ electrosynthesis. However, the role of electrolyte components is equally crucial but is significantly under‐researched. In this study, anionic surfactant *n*‐tetradecylphosphonic acid (TDPA) and its analogs are used as electrolyte additives to enhance the selectivity of the two‐electron oxygen reduction reaction. Mechanistic studies reveal that TDPA assembled over the electrode–electrolyte interface modulates the electrical double‐layer structure, which repels interfacial water and weakens the hydrogen‐bond network for proton transfer. Additionally, the hydrophilic phosphonate moiety affects the coordination of water molecules in the solvation shell, thereby directly influencing the proton‐coupled kinetics at the interface. The TDPA‐containing catalytic system achieves a Faradaic efficiency of H_2_O_2_ production close to 100% at a current density of 200 mA cm^−2^ using commercial carbon black catalysts. This research provides a simple strategy to enhance H_2_O_2_ electrosynthesis by adjusting the interfacial microenvironment through electrolyte design.

## Graphical abstract

The addition of *n*‐tetradecylphosphonic acid (TDPA) to the potassium hydroxide (KOH) electrolyte enables the improvement of the selectivity of hydrogen peroxide (H_2_O_2_) electrosynthesis. The interfacial H‐bond network is weakened, which further impacts proton transfer. TDPA involves the K^+^ solvation shell and increases the energy barrier for hydrolytic dissociation. The suitable proton supply environment improves the selectivity of H_2_O_2_ via suppressing 4e^−^ oxygen reduction.

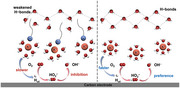

## 1 Introduction

Hydrogen peroxide (H_2_O_2_) is a valuable and fundamental chemical, which is widely used as a potential energy carrier and environmentally friendly oxidant in disinfection, chemical synthesis, etc.^[^ ^1^, ^2^, ^3^, ^4^ ^]^ The industrial synthesis of H_2_O_2_ is based on the energy‐intensive anthraquinone method, which suffers from large amounts of chemical waste and safety peril.^[^ ^5^, ^6^, ^7^ ^]^ Thus, it is vital and urgent to develop a method of H_2_O_2_ production with less energy consumption, production costs, and safety issues. Recently, electrocatalytic oxygen reduction reaction (ORR) through a selective two‐electron (2e^−^) pathway has provided a potential route for H_2_O_2_ industrial synthesis.^[^ ^8^, ^9^, ^10^, ^11^ ^]^ Its advantages include mild reaction conditions, renewable electricity input without CO_2_ emissions, high energy conversion efficiency, and green reaction precursors.^[^ ^12^, ^13^, ^14^, ^15^ ^]^ However, ORR tends to be kinetically slow and less selective due to the 4e^−^ competition reaction.

The latest efforts have been mostly focused on catalyst design.^[^ ^14^, ^16^, ^17^ ^]^ Previous reports suggest that some noble metal catalysts, including Pd, Au, and Ag, exhibit small overpotentials and high selectivity during the ORR.^[^ ^18^, ^19^, ^20^ ^]^ However, their use on a wide scale is limited by economic constraints. In contrast, no‐metal carbon‐based catalysts are advantageous due to their low cost, high conductivity, tunable physicochemical properties, and abundant reserves.^[^ ^21^, ^22^, ^23^ ^]^ Most carbon‐based electrocatalysts are more effective in alkaline than acidic conditions. Nevertheless, H_2_O_2_ is deprotonated (p*K*_a_ > 11) and easily degraded in alkaline solutions, leading to a loss in selectivity in alkaline solutions.^[^ ^24^, ^25^ ^]^ Addressing the electrochemical dissociation of H_2_O_2_ to H_2_O may be an urgent challenge to resolve this H_2_O_2_ selectivity–activity dilemma.

It is well known that electrocatalytic reactions occur in nanoscale space at the electrified electrode–electrolyte interface.^[^ ^18^, ^26^ ^]^ Both the catalyst and the electrolyte are key components of research for inert small‐molecule electrocatalysis. Understanding and controlling the electrolyte components is critical to elucidating reaction mechanisms and improving catalytic performance.^[^ ^27^, ^28^, ^29^ ^]^ Recent studies have shown that alkali metal cations,^[^ ^30^, ^31^, ^32^ ^]^ organic molecules,^[^ ^33^, ^34^ ^]^ and surfactants^[^ ^35^, ^36^, ^37^ ^]^ are commonly used as additives to regulate the electrode–electrolyte interface. For instance, Duan and co‐workers used cetyltrimethylammonium cation (CTA^+^)‐modified carbon paper to modulate reactivity and product stereoselectivity through dipolar and hydrophobic interactions, respectively.^[^ ^38^ ^]^ The small organic molecule dimethyl sulfoxide as a cationic solvation layer modifier can inhibit the hydrogen precipitation reaction by reducing the interfacial water molecule density and the relative content of the proton source.^[^ ^33^ ^]^ Under alkaline conditions, the proton source for ORR is primarily water. Altering the water activity can impact the proton‐coupled electron transfer (PCET) process of ORR and, consequently, its selectivity.^[^ ^34^, ^39^ ^]^ Taken together, we hypothesized that the long hydrophobic alkyl chains of anionic surfactants modulated the interfacial water concentration and that the hydrophilic head group acted on the cationic solvation shell. The reconstructed interfacial water environment affected the PCET process at the electrode–electrolyte interface, thereby suppressing the 4e^−^ ORR and enhancing 2e^−^ ORR activity.

In this work, we designed a catalytic system with high selectivity and stability by selecting different alkyl chain length phosphonic acid anionic surfactants as electrolyte additives. The carbon black (CB) was used as a model catalyst. Electrochemical impedance spectroscopy (EIS) and in situ attenuated total reflection surface‐enhanced infrared absorption spectroscopy (ATR‐SEIRAS) demonstrated the changes in the aqueous interfacial environment after the addition of *n*‐tetradecylphosphonic acid (TDPA). It created a proper proton providing microenvironment for highly selective ORR to H_2_O_2_.^[^ ^40^ ^]^ Molecular dynamics (MD) simulations demonstrated the involvement of TDPA in the K^+^ solvation shell and altered the water orientation structure. The experimental results were in good agreement with the simulations. The system was applied to a flow cell, resulting in a Faradaic efficiency (FE) close to 100% at a current density of 200 mA cm^−2^. This strategy for regulating the microenvironment will offer significant potential for future modulation of electrocatalytic reaction selectivity. The charged interface assembled by TDPA could suppress water transfer and build a suitable proton supply environment for highly selective ORR to H_2_O_2_. The understanding and regulation of the interfacial environment can be generalized to other electrochemical reactions in aqueous electrolytes.

## 2 Results and Discussion

### 2.1 Electrochemical ORR Performance of Carbon Catalysts in Potassium Hydroxide Electrolytes with and without TDPA

**Figure 1:**
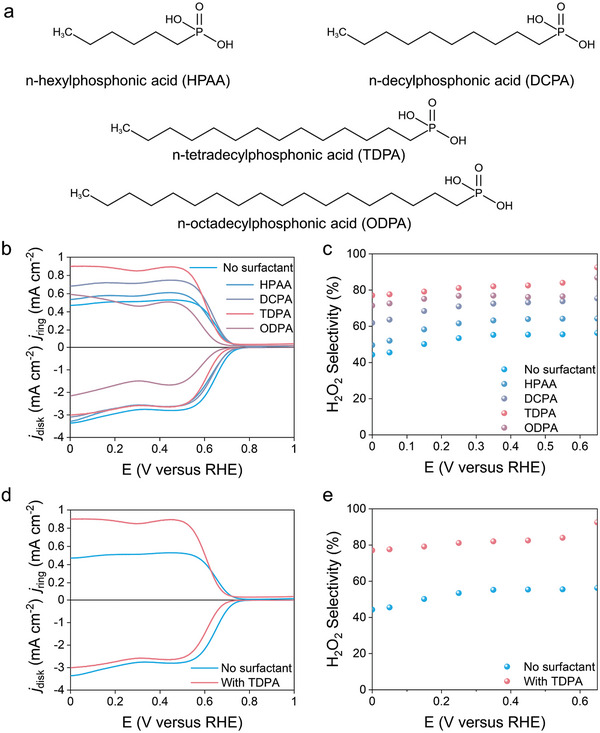
Electrochemical ORR performance. a) Structural formulas of TDPA and its analogs. b) LSV curves of CB catalyst by RRDE at a scan rate of 5 mV s^−1^ in 0.1 m KOH electrolyte and 0.1 m KOH electrolytes with HPAA, DCPA, TDPA, and ODPA. The risk current density (*j*_ring_) represented the H_2_O_2_ oxidation current. c) H_2_O_2_ selectivity in corresponding electrolytes. d) LSV curves of CB catalyst by RRDE in 0.1 m KOH electrolytes with and without TDPA. e) H_2_O_2_ selectivity in 0.1 m KOH electrolytes with and without TDPA. The surfactants were added at a concentration of 0.2 mm under the above‐mentioned experimental conditions.

To evaluate ORR activity and selectivity with the addition of surfactants, the reaction used CB as a model catalyst using a rotating ring‐disk electrode device (RRDE) at 1600 rpm. The electrolytes were O_2_‐saturated 0.1 m potassium hydroxide (KOH) and 0.1 m KOH with additives. The ring electrode was held at 1.2 V versus a reversible hydrogen electrode (RHE, *V*_RHE_) to monitor the H_2_O_2_ selectivity. We used several types of phosphonate anionic surfactants TDPA and its analogs as electrolyte additives. These surfactants include *n*‐hexylphosphonic acid (HPAA), *n*‐decylphosphonic acid (DCPA), TDPA, and *n*‐octadecylphosphonic acid (ODPA), the molecule structure, as shown in **Figure** **1**a. The ORR polarization curves of CB in RRDE in 0.1 m KOH electrolytes with the addition of surfactants of different alkyl chain lengths, together with the H_2_O_2_ current density detected by the Pt ring, are shown in Figure 1b. The corresponding H_2_O_2_ selectivity is plotted in Figure 1c. With the incorporation of different surfactants, both ring current density and H_2_O_2_ selectivity were significantly changed. The 0.1 m KOH electrolyte with TDPA showed preferable 2e^−^ ORR selectivity. Within a wider potential window from 0 to 0.65 V_RHE_, the KOH electrolytes with TDPA and ODPA exhibit higher selectivity than with HPAA and DCPA. Longer alkyl chains appear to be more favorable to the 2e^−^ ORR pathway. The ORR activity is reduced with the addition of ODPA, possibly due to the impact of the longer alkyl chain length on the density of the assembly layer. The molecular packing density affects the transport of gas to the catalyst surface. Thus, to optimize the selectivity of 2e^−^ ORR, TDPA was subsequently chosen to explore the impact of alkylphosphonic acid molecular additives on the system.

In addition, we further tested the linear sweep voltammetry (LSV) of the electrolyte systems with different concentrations of TDPA (Figure S3, Supporting Information). The KOH electrolyte with TDPA notably enhances both ring current and H_2_O_2_ selectivity. It was found that the addition of 0.2 mm TDPA exhibited the best performance. Figure 1d,e shows the LSV and H_2_O_2_ selectivity of the 0.2 mm TDPA electrolyte system. In a wide potential window from 0 to 0.65 V_RHE_, the KOH electrolyte with TDPA resulted in a significant increase in selectivity compared to the KOH electrolyte. Moreover, the electron transfer number in the KOH electrolyte with TDPA (2.3–2.4) is close to the electron transfer number of the theoretical 2e^−^ ORR (Figure S4, Supporting Information). It can effectively promote the 2e^−^ ORR pathway, especially under high bias potential (<0.35 V_RHE_). The selectivity of H_2_O_2_ was increased from 45% to 85%. The classical two‐step ORR is evident in the LSV curve of CB: the initial step involves the reduction of oxygen to hydrogen peroxide (from 0.75 to 0.35 V_RHE_), followed by a subsequent H_2_O_2_ decomposition step (<0.35 V_RHE_).^[^ ^41^ ^]^ Here, we believe that the TDPA molecule effectively inhibits the H_2_O_2_ decomposition step. Four different carbon‐based catalysts including carbon nanotubes (CNTs), reduced graphene oxide (rGO), acetylene black (ACET), and cochineal black (KB) were selected to further evaluate the performance of the KOH electrolytes with and without TDPA (Figures S6–S9, Supporting Information). Similar phenomena were observed on other carbon catalysts (Figures S10–S13, Supporting Information). This indicates that surfactant‐modified electrolytes exhibit universal applicability for boosting the H_2_O_2_ selectivity of carbon‐based catalysts.

### 2.2 Kinetics Investigation of Electrochemical Processes of ORR

**Figure 2:**
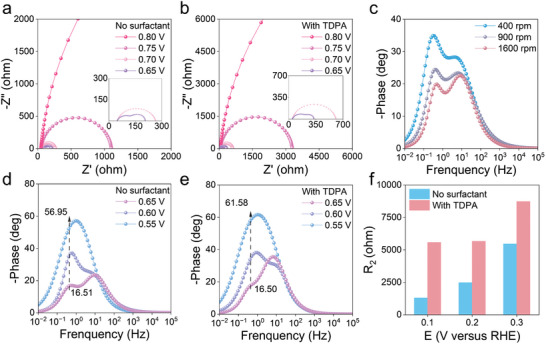
Reaction kinetics analysis by electrochemical impedance spectroscopy. a) Nyquist plots for 0.1 m KOH electrolyte from 0.8 to 0.7 V_RHE_. b) Nyquist plots for 0.1 m KOH electrolyte with 0.2 mm TDPA from 0.8 to 0.7 V_RHE_. c) Bode plots of 0.1 m KOH electrolyte at different rotational speeds at 0.55 V_RHE_. d) Bode plots of 0.1 m KOH electrolyte from 0.65 to 0.55 V_RHE_. e) Bode plots of 0.1 m KOH electrolyte with 0.2 mm TDPA from 0.65 to 0.55 V_RHE_. f) The resistance value comparison of 0.1 m KOH electrolytes with and without 0.2 mm TDPA from 0.3 to 0.1 V_RHE_.

EIS was utilized to track the electrochemical processes of ORR at various potentials. The equivalent circuit models for the EIS measured in 0.1 m KOH with and without 0.2 mm TDPA are depicted in Figure S14 (Supporting Information).^[^ ^42^ ^]^ Prior to reaching 0.75 V_RHE_, the equivalent resistance is significantly high, indicating weak charge transfer. After reaching 0.75 V_RHE_, the charge‐transfer resistance *R*_1_ suddenly decreases. This indicates that the ORR process occurs. According to the LSV curve in Figure 1d, the reaction occurs at 0.75 V_RHE_. With increasing bias potential from 0.75 to 0.65 V_RHE_, *R*_1_ decreases from 1056 to 98.55 Ω. (**Figure** **2**a; Figure S17, Supporting Information). At this stage, 2e^−^ ORR predominates, and the high H_2_O_2_ selectivity is maintained. The resistance becomes larger after the addition of TDPA (Figure 2b), which is due to the alkyl chain decreasing the local dielectric constant.^[^ ^40^, ^42^ ^]^

A new arc appears in the Nyquist plots from 0.65 to 0.55 V_RHE_. According to the bode plot in Figure 2c at different rotation speeds, the phase angle amplitude (*Ø*_peak_) of the low frequency decreases notably more than that of the high frequency with increasing rotation speed. This suggests that the electron‐transfer process corresponding to the low frequency is affected by mass transfer. For the pure KOH electrolyte, the low‐frequency *Ø*_peak_ ranges from 16.5° to 56.95° over the range of 0.65–0.55 V_RHE_, while after the addition of TDPA, it extends from 16.5° to 61.58°. In addition, the charge‐transfer resistance *R*_2_ associated with the mass transfer increases from 205.1 to 435.5 Ω at 0.6 V_RHE_. These indicate that the TDPA affects the mass‐transfer process and thus the charge‐transfer process (Figure 2d,e). As shown in the LSV curve shown in Figure 1d, the response exhibits an inflection point at 0.4 V_RHE_. From 0.4 to 0 V_RHE_, the selectivity of H_2_O_2_ in pure KOH electrolyte decreases, and *R*_2_ decreases from 5461 to 1302 Ω with increasing bias potential. These results suggest that rapid electron transfer is detrimental to the 2e^−^ ORR, which favors the further reduction of hydrogen peroxide, primarily a 4e^−^ ORR process.

The KOH electrolyte with TDPA apparently maintained high 2e^−^ ORR selectivity. Figure 2f and Figure S17 (Supporting Information) compare the *R*_2_ values from 0.3 to 0.1 V_RHE_ with and without TDPA. The *R*_2_ increases significantly in KOH electrolyte with TDPA compared to without TDPA. And the rate of decrease in *R*_2_ slows down as the bias voltage increases. The results suggest that TDPA molecules play a crucial role in modulating the charge‐transfer process, thereby preventing excessive reduction of H_2_O_2_. At this stage, charge‐transfer resistance is predominantly influenced by mass transfer. Hence, we propose that TDPA mainly affects the mass transfer of water molecules. Subsequent investigations included EIS tests on the hydrogen evolution reaction interval under the N_2_ atmosphere (Figure S18, Supporting Information). The KOH electrolyte with TDPA led to a substantial increase in resistance. Since water serves as the primary source of protons under alkaline conditions, this phenomenon suggests that TDPA indeed influences the content of water molecules.

### 2.3 In Situ Spectroscopy Investigation of the Electrode–Electrolyte Interface

**Figure 3:**
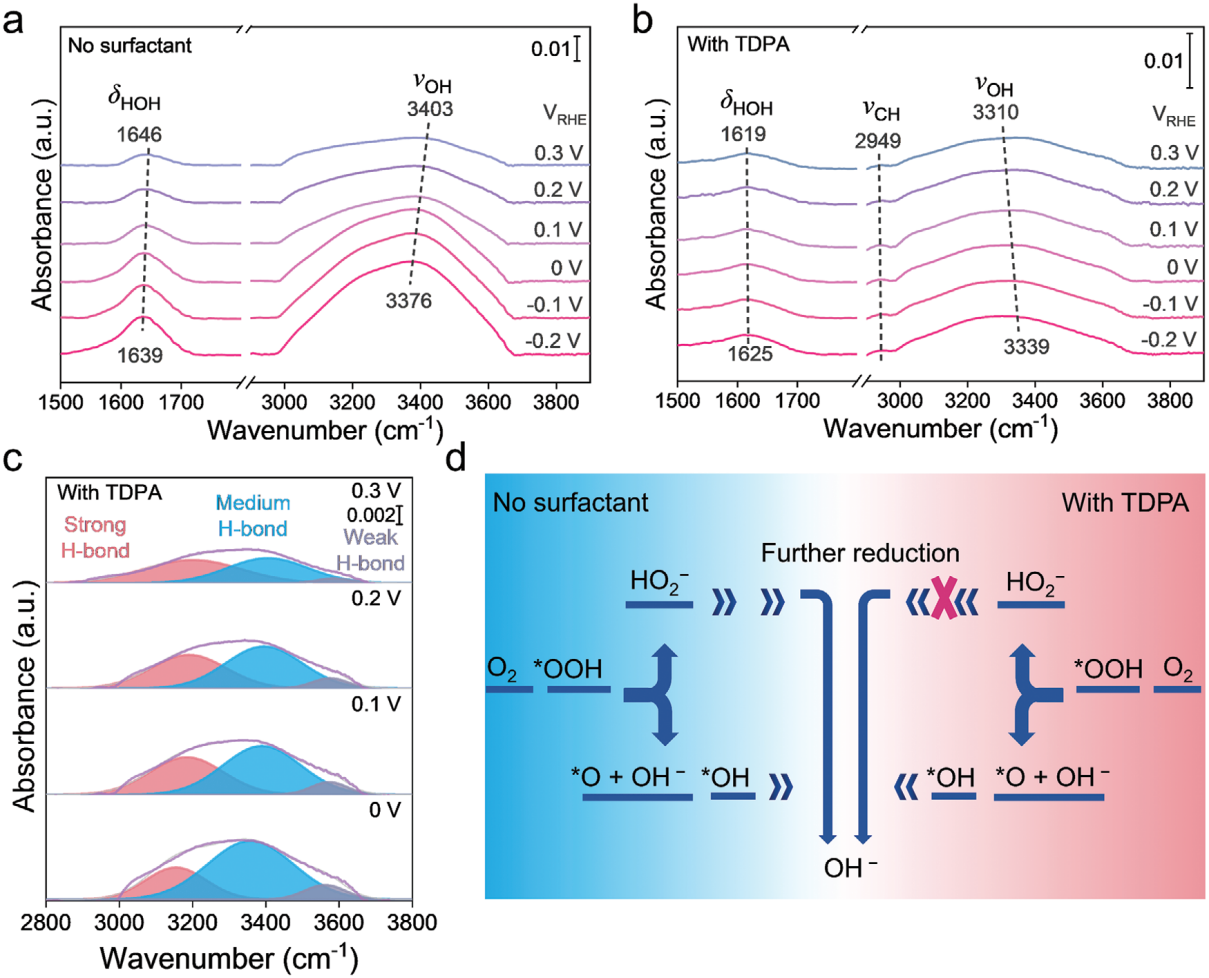
In situ spectroscopy investigation of the electrode–electrolyte interface. a) In situ ATR‐SEIRAS spectra of 0.1 m KOH electrolyte from 0.3 to −0.2 V_RHE_. b) In situ ATR‐SEIRAS spectra of 0.1 m KOH with 0.2 mm TDPA electrolyte from 0.3 to −0.2 V_RHE_. c) In situ ATR‐SEIRAS spectra of *ν*‐OH at the interface of 0.1 m KOH with 0.2 mm TDPA electrolyte from 0.3 to 0 V_RHE_. d) Reaction path diagram before and after the addition of 0.2 mm TDPA.

To elucidate the impact of TDPA at the electrode–electrolyte interface, we conducted further exploration using in situ ATR‐SEIRAS from open circuit potential (0.8 V_RHE_) to −0.2 V_RHE_ (Figures S19 and S20, Supporting Information). The peak from 2800 to 3800 cm^−1^ is attributed to the O─H stretching vibration (*ν*‐OH) of water, while the peak near 1630 cm^−1^ is attributed to the H─O─H bending vibration (*δ*‐HOH) (**Figure** **3**a,b). The peak at 2949 cm^−1^ could be assigned to the symmetric C─H vibrational bands (*ν*‐CH) of the ─CH_3_ of TDPA (Figure 3b). The intensity of the *ν*‐CH initially increases to 0.1 V_RHE_ and then decreases as the potential becomes negative. This indicates that the assembly process of TDPA at the interface initially enriches and then gradually moves away as the potential becomes negative. The intensities of the *ν*‐OH and *δ*‐HOH exhibit significant increases within increasing bias potential, indicating the continuous enrichment of water at the electrified interface. Furthermore, a redshift of the vibrational frequency of the *ν*‐OH from ≈3403 to ≈3376 cm^−1^ was observed as the applied potentials shifted negatively from 0.3 to −0.2 V_RHE_ (Figure 3a). This phenomenon indicates an enhancement of the hydrogen‐bond (H‐bond) network between the interfacial water in response to more negative surface charge.

It can be seen that the vibrational frequency of the *ν*‐OH is blueshifted from ≈3310 to ≈3339 cm^−1^ in the KOH electrolyte with TDPA. Based on previous studies, the *ν*‐OH vibration mode between 2800 and 3800 cm^−1^ can be deconvoluted into three types with different H‐bond strengths: ≈3200 cm^−1^ (strong H‐bond), ≈3400 cm^−1^ (medium H‐bond), and ≈3600 cm^−1^ (weak H‐bond).^[^ ^43^, ^44^ ^]^ Gaussian fitting was utilized to divide the *ν*‐OH vibrational peaks at 2800–3800 cm^−1^. Figure 3c shows that the relative proportions of strong H‐bond decreased and weak H‐bond increased as the potential became negative in KOH with TDPA electrolyte. These results suggest a weakened H‐bond in the electrical double layer (EDL) further structure with the introduction of TDPA results.^[^ ^34^, ^45^ ^]^ As a result, the proton transfer from bulk electrolyte to electrode–electrolyte interface is impeded.

Combined with EIS and spectroscopic analyses, the drop in H_2_O_2_ selectivity of the pure KOH electrolyte after 0.35 V_RHE_ can be attributed to the increase in the content of interfacial water. In a H‐bond network formed by water molecules, protons are transferred through the process of H‐bond breaking and reorganization.^[^ ^46^, ^47^ ^]^ Therefore, proton transfer is faster with the ordered H‐bond network. This leads to the further hydrogenation of H_2_O_2_ to H_2_O. With the addition of TDPA, the interfacial water content decreased due to the hydrophobic effect induced by the long alkyl chains of TDPA molecules. In addition, TDPA molecules alter the H‐bond structure of the interfacial water, which further impacts proton transfer. This ultimately inhibits the competitive 4e^−^ reaction and promotes the 2e^−^ reaction to generate H_2_O_2_ (Figure 3d).

### 2.4 TDPA Influence on K^+^ Solvation Structure: MD and Quantum Chemistry Insights

To further understand the surfactant‐tuned mechanism of H_2_O_2_ electrosynthesis, we explored the effects caused by the head group of the TDPA molecule. Figure S25 (Supporting Information) displays the cyclic voltammetry curves in KOH electrolyte with different concentrations of TDPA. It can be observed that the change in the EDL is not significant. Another electrochemical active area (ECSA) test was conducted (Figure S26, Supporting Information). The ECSA of the electrode before and after the addition of TDPA was found to be 1.16 and 1.1 mF cm^−2^, respectively, with no significant changes. By comparing the X‐ray photoelectron spectroscopy (XPS) spectra of P 2p of the TDPA molecules and the gas diffusion electrode (GDE) in TDPA‐containing system (Figure S28, Supporting Information), we found that there was no peak shift in XPS spectra, suggesting that the detection of P element in GDE after biased might be due to residual electrolyte, and there was no chemical binding between TDPA and electrode surface.^[^ ^48^ ^]^ These suggest that TDPA molecules did not adsorb onto the electrode surface.

**Figure 4:**
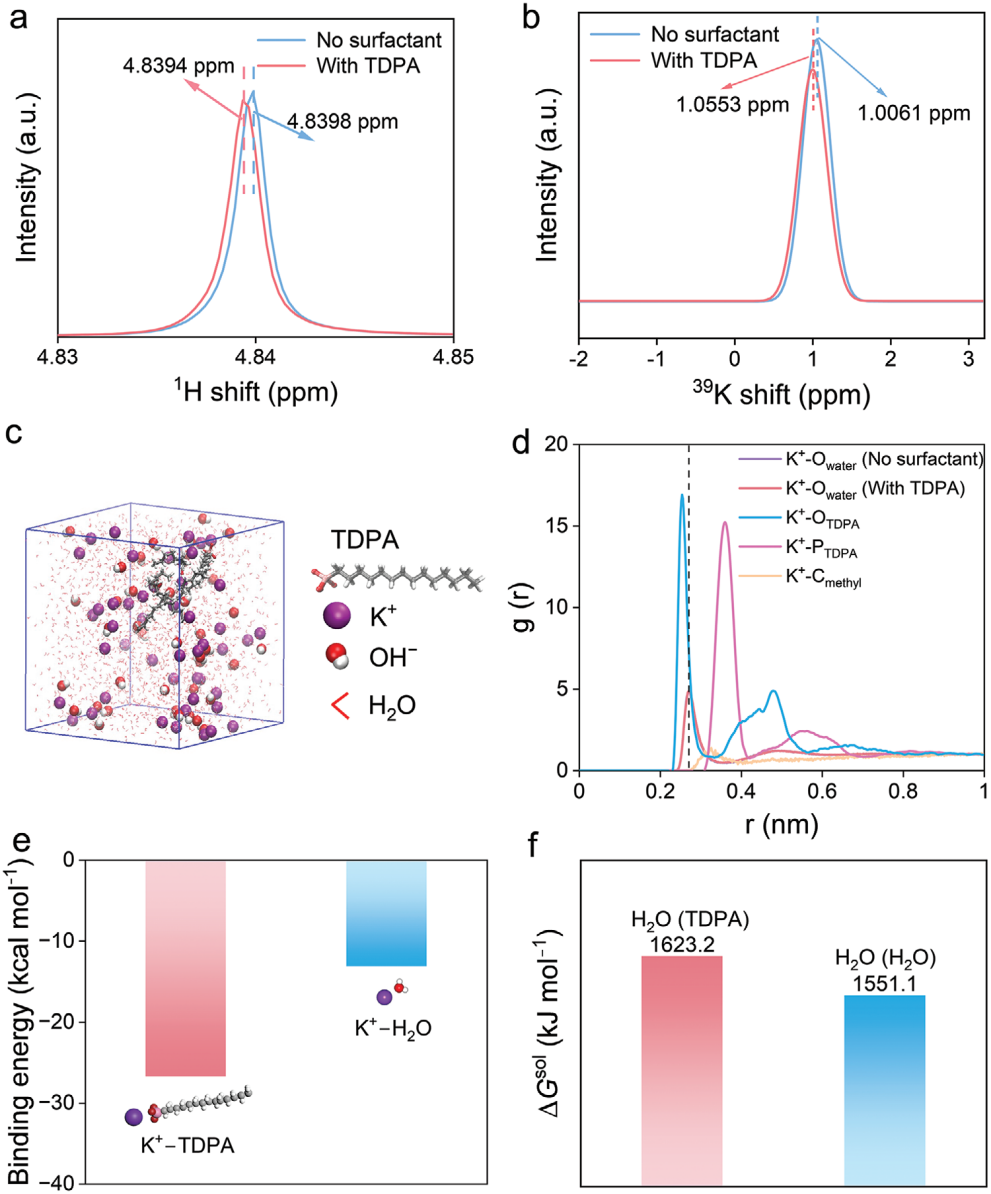
Molecular dynamics simulation and quantum chemistry calculation. a) ^1^H NMR spectra of the KOH electrolytes with and without TDPA. b) ^39^K NMR spectra of the KOH electrolytes with and without TDPA. The concentration of KOH in the NMR test was 0.1 m, and the added TDPA concentration was 0.2 mm. c) Snapshot of the KOH electrolyte with TDPA from MD simulation. d) RDF of K^+^─O_water_, K^+^─O_TDPA_, K^+^─P, and K^+^─C_methyl_ in KOH electrolytes with and without TDPA. e) The binding energy of K^+^─TDPA and K^+^─H_2_O from MD simulation. f) Free energy of the H_2_O (H_2_O) and H_2_O (TDPA) clusters.

^1^H and ^39^K nuclear magnetic resonance (NMR) spectroscopy were used to characterize the electrolyte. The KOH electrolyte with TDPA caused a chemical shift of ^1^H of water toward the high field compared the KOH electrolyte, along with a broadening of the peak shape in **Figure** **4**a. This shift is attributed to the shielding effect of the formation of hydrogen bonding interactions between TDPA and water molecules.^[^ ^49^ ^]^ Furthermore, ^39^K NMR spectroscopy was used to investigate the coordination environment of cations in the KOH electrolyte (Figure 4b). The addition of TDPA caused a shift of the potassium spectrum to the lower field due to a weakening of the shielding effect on K^+^. This change in the solvation structure of K^+^ was tentatively attributed to the involvement of TDPA molecules in the solvation shell layer of it.^[^ ^50^ ^]^ Based on the above results, it is tentatively concluded that the TDPA molecules entered the solvated shell layer of K^+^ and assembled at the electrode interface.

MD simulations were employed to further demonstrate the effect of TDPA on the solvation structure of K^+^. Figure 4c displays snapshots of the MD simulations for the 0.1 m KOH electrolyte with and without TDPA molecule. The radial distribution function (RDF) analysis reveals a peak at 0.272 nm for K─O_water_. It is attributed to the H_2_O molecule in the first solvation shell of K^+^ (Figure 4d). In the presence of TDPA, the sharp peak of K^+^─O_TDPA_ at 0.252 nm is observed. This is within the first solvation shell layer of K^+^. The position of the first RDF peak for K^+^─P_TDPA_ is 0.366 nm. The results indicate that the TDPA molecule is incorporated into the first solvation shell of K^+^. The oxygen in the TDPA molecule coordinates with K^+^.

Furthermore, we used the quantum chemistry calculations to calculate the binding energy of different solvation structures (Figure 4e). The binding energy of TDPA with K^+^ is −27.24 kcal mol^−1^, significantly lower than that of H_2_O with K^+^ of −13.13 kcal mol^−1^. It suggests that the coordination structure of K^+^ with TDPA has higher stability. Density functional theory was used to calculate the water dissociation energy before and after adding TDPA molecules. This method illustrates the energy barrier required to deprotonate H_2_O in the clusters. The free energy of the H_2_O clusters formed after introducing TDPA molecules was 1623.2 kJ mol^−1^, significantly higher than that of the H_2_O clusters (1551.1 kJ mol^−1^). The TDPA molecule forms H‐bonds with water, altering the H‐bond structure and increasing the water dissociation energy barrier. And the enrichment of TDPA at the interface causes K^+^ to aggregate at the interface (Figure S30, Supporting Information). These results can decrease the H_2_O density at the interface and the relative content of proton suppliers. The suitable proton supply environment hinders excessive hydrogenation of the ORR, thus improving the selectivity of 2e^−^ ORR.

### 2.5 Evaluation of H_2_O_2_ Electrosynthesis Performance in Flow Cell

**Figure 5:**
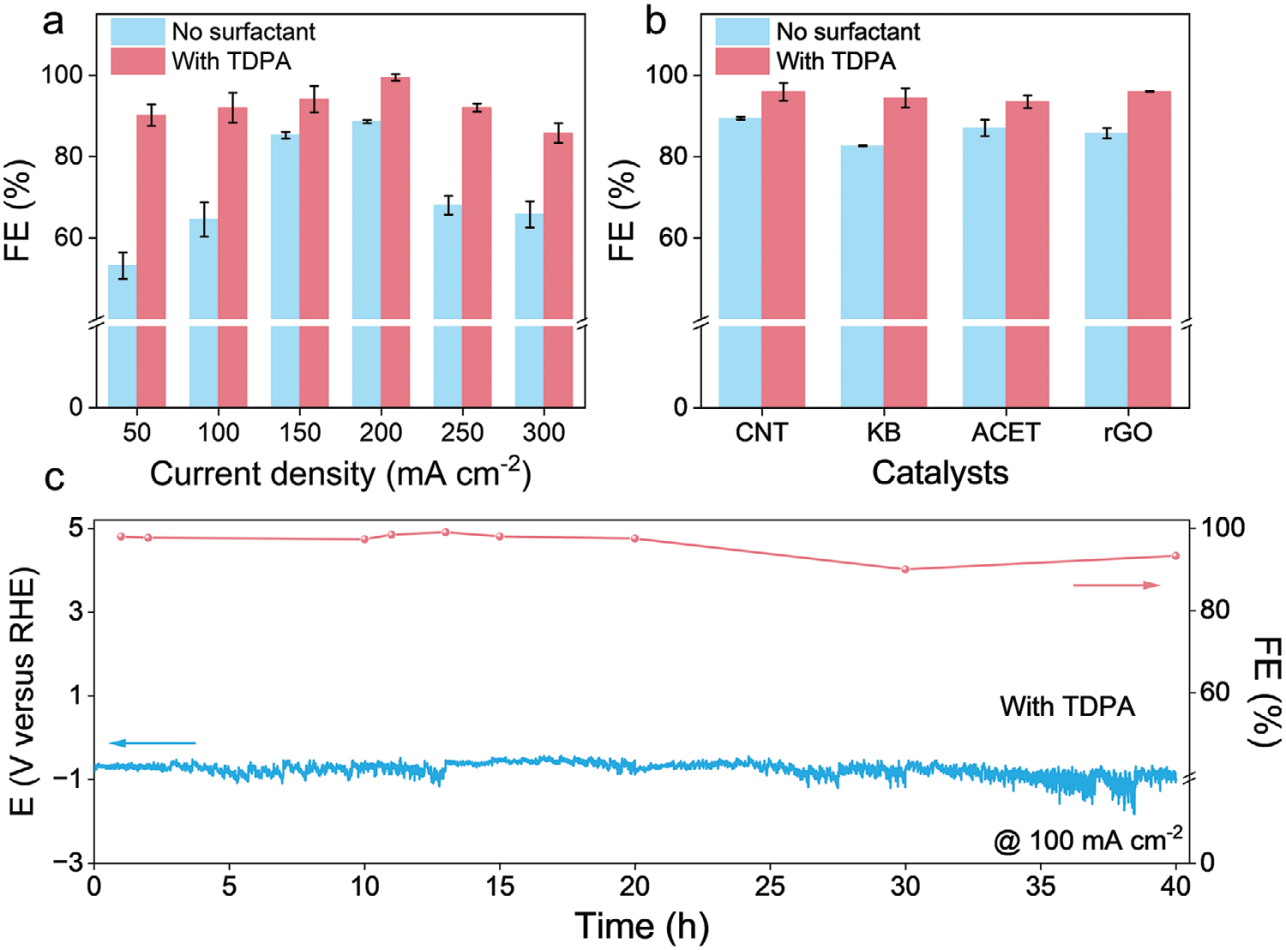
Evaluation of H_2_O_2_ electrosynthesis performance in a flow cell. a) Comparison of H_2_O_2_ FE in 0.1 m KOH electrolytes with and without 0.2 mm TDPA at different current densities. b) Comparison of H_2_O_2_ FE in 0.1 m KOH electrolytes with and without 0.2 mm TDPA with different carbon‐based catalysts at 200 mA cm^−2^ current density. c) Stability of KOH–TDPA electrolyte at 100 mA cm^−2^ current density. The error bars represent the standard deviations of three independent measurements.

Based on the RRDE screening test, we found that the KOH electrolyte with TDPA can maintain high H_2_O_2_ selectivity. We further evaluated the 2e^−^ ORR performance of our electrolyte system using a flow cell reactor. The flow cell was used to facilitate O_2_ gas diffusion and achieve large‐scale ORR current densities. The catalyst employed was carbon black on the GDE. The cathode electrolyte and the anode electrolyte flow rates were 10 and 20 mL min^−1^, respectively. The cathode contained 100 mL of circulating electrolyte. The oxygen supply rate was set at 20 sccm min^−1^. The reference electrode used was a Hg/HgO electrode, and a platinum mesh was used as the counter electrode in the anode chamber of the oxygen evolution reaction. The concentration of generated H_2_O_2_ was determined using the cerium sulfate standard curve method (Figure S32, Supporting Information).^[^ ^51^ ^]^ The corresponding FE was calculated from the generated H_2_O_2_ concentration and the applied current. **Figure** **5**a shows the H_2_O_2_ FE of the KOH electrolytes with and without TDPA at various current densities. The FE and yield of H_2_O_2_ in a 0.1 m KOH electrolyte were low for GDE. The H_2_O_2_ FE reached a maximum of 88% at 200 mA cm^−2^. In contrast, in the KOH electrolyte with 0.2 mm TDPA, the H_2_O_2_ FE at each current density was significantly increased, even approaching 100% at 200 mA cm^−2^. There is still ≈85% H_2_O_2_ FE at a current density of 300 mA cm^−2^. A substantial increase in the yield rate was also observed (Figure S33, Supporting Information). Moreover, the performance of four different carbon‐based catalysts, CNT, rGO, KB, and ACET, was investigated with and without 0.2 mm TDPA in a 0.1 m KOH electrolyte at 200 mA cm^−2^ (Figure 5b). The addition of TDPA significantly enhanced H_2_O_2_ FE, indicating the universality of the KOH–H_2_O–TDPA electrolyte system. A 40 h stability test was also conducted. It was found that the voltage variations during the test were small, and the H_2_O_2_ FE could be maintained above 90% over the testing period. We further investigated the effect of the TDPA on H_2_O_2_ electrosynthesis in 0.05 m K_2_SO_4_ in a flow cell. As shown in Figure S34 (Supporting Information), the addition of TDPA achieved the higher FE of H_2_O_2_ than that of the systems without TDPA in a wide current window, indicating that the surfactant modulation mechanism is conducive to H_2_O_2_ electrosynthesis in neutral electrolytes.

## 3 Conclusion

In summary, we have demonstrated a facile strategy to enhance 2e^−^ ORR selectivity by manipulating the electrode–electrolyte interface. Experimental data suggest that the introduction of TDPA repels interfacial water, modulates the H‐bond in the EDL, and then suppresses proton‐transfer kinetics. This reconfiguration of the interfacial water environment had a direct impact on the PCET process at the electrode–electrolyte interface, leading to the suppression of 4e^−^ ORR and the promotion of 2e^−^ ORR. Notably, we achieved the FE of H_2_O_2_ production approaching 100% at a current density of 200 mA cm^−2^. This work is also applicable to flow cell and is universal to other carbon‐based catalysts. These findings underscore the potential of our approach to significantly advance electrocatalysis.

## 4 Experimental Section

### Materials

Carbon black was supplied by Macklin (cabot vulcan XC‐72R). Phosphonate anionic surfactants with different alkyl chain lengths: HPAA (98%), DCPA (98%), TDPA (98%), ODPA (97%), and KOH (99.99%) were purchased from Macklin. The cerous sulfate standard solution (Ce(SO_4_)_2_, 0.1 mol L^−1^) was supplied by Bolinda. The water mentioned in this work was deionized water (18.2 MΩ cm). GDE was supplied by SGL (Sigracet 28 BC).

### Electrochemical Measurement

To prepare the catalyst ink, first 5 mg of CB was sonicated in 490 µL of ethanol and 490 µL of ultrapure water for 5–10 min. Then, 20 µL of Nafion solution (5.0 wt%, Macklin) was added to the sonicated solution and was sonicated for another 30 min to obtain the catalyst ink. About 5 µL of catalyst ink was taken and it was evenly spread on the glassy carbon electrode. It was allowed to dry naturally. The catalyst loading was 0.1 mg cm^−2^.

The electrochemical tests were performed in a three‐electrode system controlled by electrochemical workstations (CHI760E, CHI1140D, CH Instruments). The system consisted of a reference electrode, a counter electrode, and a working electrode. Specifically, an Ag/AgCl (RREF0024, Pine Research Instrumentation) as a reference electrode with a salt bridge, a graphite counter electrode, and a catalyst‐loaded RRDE (AFE7R9GCPT, Pine Research Instrumentation) as work electrode were utilized in the RRDE. The flow cell utilized the Hg/HgO reference electrode, platinum mesh as the counter electrode, and a gas diffusion electrode as the working electrode. The RRDE included a glassy carbon disk electrode (disk area = 0.2475 cm^−2^) and a Pt ring electrode (ring area = 0.1866 cm^−2^) with a theoretical collection efficiency (*N*) of 0.37. The electrolyte was exposed to oxygen for at least 30 min before undertaking electrochemical tests. The electrochemical experiments were performed in 0.1 m KOH saturated with oxygen at room temperature (RT). Before tests, at the range of 0–1 V_RHE_, the cyclic voltammetry tests were done for 20 cycles at a scan rate of 50 mV s^−1^. For LSV tests, the scan rate was 5 mV s^−1^, the rotation rate of the working electrode was set at 1600 rpm, and a constant potential of 1.2 V_RHE_ was applied on the ring electrode to detect the generated H_2_O_2_. All potentials were calibrated to the RHE reference scale using the formulas as follows

(1) $$E_{\mathrm{RHE}}=E_{\mathrm{Ag}/\mathrm{AgCl}}+0.0592\;\times \;\mathrm{pH}\;+0.197$$

Equations (2) and (3) were used to calculate the electron transfer number (*n*) and H_2_O_2_ selectivity (%), where *I*_r_ is the ring current, and *I*_d_ is the disk current

(2) $$H_{2}O_{2}\left(\%\right)=\frac{200\times I_{r}}{\left|I_{d}\right|\times N+I_{r}}$$

(3) $$n=4\times \frac{\left|I_{d}\right|}{\left|I_{d}\right|+\frac{I_{r}}{N}}$$

### Electrochemical Impedance Spectroscopy Measurement

The electrolytes utilized in the experiments were 0.1 m KOH and 0.1 m KOH electrolyte containing 0.2 mm TDPA. The test frequency ranged from 10^−2^ to 10^5^ Hz, with a voltage range of 0–0.9 V_RHE_. The RRDE was rotated at a speed of 1600 rpm, and changes in the dominant process during the ORR process were detected in real time. The Bode plot processes were determined by measuring the in situ electrochemical impedance spectra at rotational speeds of 400, 900, and 1600 rpm. The EIS data were fitted using ZView2. *R*_s_ is the solution resistance, which was not specifically discussed here. *R*_1_ or *R*_2_ is the interface resistance of the ORR.

### Performance Test in a Flow Cell

During the experiment, a 0.1 m KOH solution was used as the anodic electrolyte in the flow cell, while the cathodic electrolyte was a 0.1 m KOH solution with the addition of 0.2 mm TDPA. The anodic flow rate was set at 20 mL min^−1^, and the cathodic flow rate was set at 10 mL min^−1^. A proton membrane Nafion 117 was utilized as the diaphragm. The reaction feedstock consisted of high‐purity oxygen with a fixed flow rate of 20 sccm. Oxygen was supplied to the three‐phase interface between the catalyst and the cathode electrolyte via a gas diffusion electrode for the 2e^−^ ORR reaction. The anode employed a platinum mesh for OER. For the three‐electrode flow‐cell setup, all potentials measured against Hg/HgO were converted to the RHE scale using the formulas (Equation (4))

(4) $$E_{\mathrm{RHE}}=E_{\mathrm{Hg}/\mathrm{HgO}}+0.0592\;\times \;\mathrm{pH}\;+0.098$$

## Conflict of Interest

The authors declare no conflict of interest.

## Author Contributions

W.S. and L.T. contributed equally to this work. H.L.J. and C.Z.L. conceived the idea, designed the experiments, and wrote the draft. W.S. performed electrochemical measurements and data analysis. L.T. carried out MD simulation and analyzed the calculation results. W.X.G., X.D.S., and W.F.Z. contributed to the analysis of in situ ATR‐SEIRAS data. Y.F. and L.D. contributed to the analysis of EIS data. All authors discussed the results and commented on the manuscript.

## Supporting information

Supporting Information

## Data Availability

The data that support the findings of this study are available from the corresponding author upon reasonable request.
