# Supplementary material for: Anionic Surfactant‐Modulated Electrode–Electrolyte Interface Promotes H2O2 Electrosynthesis
Source: Adv Sci (Weinh). 2024 Jul 25;11(36):2405474. doi: 10.1002/advs.202405474 (PMC11423143; doi:10.1002/advs.202405474)
Supplement: Supplementary file 1 — Supporting Information [file ADVS-11-2405474-s001.docx]

Supporting Information

Anionic Surfactant-modulated Electrode-electrolyte Interface Promotes H_2_O_2_ Electrosynthesis

*Wen Sun, Lei Tang, Wangxin Ge, Yu Fan, Xuedi Sheng, Lei Dong, Wenfei Zhang, Hongliang Jiang***, and Chunzhong Li**

**Experimental Methods**

**Characterization**

Transmission electron microscopy (TEM) images were obtained using a JEM-2100 operating at an accelerating voltage of 200 kV. Scanning electron microscopy (SEM) images were collected using a Hitachi S-4800 microscope with an accelerating voltage of 10 kV. NMR spectra were collected on Bruker ASCEND600. In situ attenuated total reflection surface-enhanced infrared absorption spectroscopy (ATR-SEIRA) measurements were conducted with a Thermo-Fisher Nicolet iS20 equipped with a liquid nitrogen-cooled MCT detector and a Si attenuated total reflection (UATR) accessor.

**Hydrogen peroxide concentration detection method**

The concentration of H_2_O_2_ was detected using cerium sulfate titration and UV-Visible Absorption Spectrometry. The specific method of operation is as follows: a certain amount of the reaction product solution is mixed with a standard solution containing Ce^4+^. Based on the mechanism shown in formula (5), Ce^4+^ can be reduced to Ce^3+^ by H_2_O_2_, causing the solution to change from yellow to colorless.

$$\begin{aligned} 2\mathrm{Ce}^{4+} + H_{2}O_{2}\to\mathrm{Ce}^{3+} + O_{2}+2H^{+}\#\left( 5 \right) \end{aligned}$$

The concentration of Ce^4+^ was measured before and after titration using UV-visible spectroscopy (UV-2600, Shimadzu) at 318 nm. The concentration of H_2_O_2_ in the sampling solution was calculated based on the change in Ce^4+^ concentration. The Faraday efficiency (FE) of the 2e^−^ ORR was calculated using equation (6).

$$\begin{aligned} Faradaic Efficiency\left( \% \right)=\frac{2CVF}{Q} \times100\% \#\left( 6 \right) \end{aligned}$$

where C (mol) is the concentration of H_2_O_2_, V (L) is the volume of electrolyte, F (96485 C mol^−1^) is the Faraday constant, and Q (C) is the quantity of charge through the flow cell.

**In situ** **ATR-SEIRAS**

A polycrystalline Au nanofilm was deposited chemically onto a Si ATR-IR prism. A platinum wire and Hg/HgO were used for the counter and reference electrodes, respectively. The ATR-SEIRAS experiment was conducted with a Thermo-Fisher Nicolet iS20 equipped with a liquid nitrogen-cooled MCT detector and a Si attenuated total reflection (UATR) accessory. The spectral resolution was set to 8 cm^−1^. The data was collected with ~40 s resolution per spectrum and measured simultaneously by Chronoamperometry technique between open circuit potential (~0.8 V_RHE_) to −1.2 V_RHE_. Before the spectra were collected, the work electrodes were pre-reduced by cyclic voltammetry (0.8 V_RHE_ to 0 V_RHE_, 50 mV s^−1^, 20 cycles) to keep the catalyst in a relatively stable state. Reference spectra for the SEIRAS measurements were recorded at OCP in O_2_-saturated 0.1 M KOH electrolytes with and without 0.2 mM TDPA.

**Inductively coupled plasma optical emission spectroscopy**

ICP was performed on an Agilent 725 (Agilent, USA). Placing the sample electrodes in 0.1 M KOH electrolytes with and without TDPA with a constant potential at 0.3 V_RHE_ for 60 s. Then the electrode was charged and transferred into 100 mL ultrapure water and shaking it for 10 s, repeating the above steps 5 times to capture the K^+^ adsorbed on the surface of the electrode.

**Molecular simulations**

The molecular dynamic simulations were performed using the GROMACS code^[1]^, to gain a deeper understanding of the changes in the solvation shell upon the addition of 0.1 M TDPA in the 1 M KOH aqueous electrolyte system. All the geometric optimizations were performed using Gaussian 09 at the B3LYP/Def2-TZVP theoretical level. The force field parameters of OH^−^ and TDPA were all obtained from the CHARMM force field (Table S3). TIP3P potential model was used to describe water molecules. A total 10 ns molecular dynamic simulation run was performed for each system after energy minimization and a heating procedure to reach 300 K. The temperature and pressure were controlled using a modified Berendsen thermostat and Berendsen barostat, respectively. The sampling strategies were performed under NPT condition (300 K and 1 bar) with 2 fs time step. Details of the model components simulations are shown in Table S4.

DFT calculations were performed using Gaussian 09 to distinguish the energy profile of H_2_ evolution from water in different H-bond systems captured from MD simulations. Density functional B3LYP^[2]^ with Def2-TZVP basis set and GD3BJ^[3]^ dispersion correction were used for geometrical optimization and thermodynamic correction. The half cycle of H_2_ evolution could be described as:

$$\begin{aligned} Water/TDPA\cdot HOH + e^{-} \to Water/TDPA\cdot OH^{-} + 0.5H_{2}\#\left( 7 \right) \end{aligned}$$

The free energy for the complex of interest denoted as water/TDPA H-bond cluster in solution could be calculated according to the following expression (Table S3):

$$\begin{aligned} {\Delta G}^{sol} = {\Delta G}^{sol}\left( Water/TDPA\cdot OH^{-} \right)-{\Delta G}^{sol}\left( Water/TDPA\cdot HOH \right)\#\left( 8 \right) \end{aligned}$$

Where ${\Delta G}^{sol}$ is the free energy (kJ mol^−1^).

**Performance Evaluation of Proton Exchange Membranes.**

Scanning electron microscopy (SEM) images were collected using a Hitachi S-4800 microscope with an accelerating voltage of 10 kV.

The proton conductivity of Nafion 117 membrane was determined using the AC impedance method (CHI760E, CH Instruments). During the test, the AC perturbation amplitude was 10 mV, and the frequency range was 10^2^-10^5^Hz. Throughout the test, the Nafion 117 membrane was immersed in deionized water to ensure full wetting of the membrane. Calculate the ion conductivity of the membrane using the following formula:

$$\begin{aligned} \sigma=\frac{L}{R A}\#(9 \end{aligned})$$

Where: σ is the proton conductivity (S·cm^−1^); L is the thickness of the electrolyte membrane (cm); R is the bulk resistance of the electrolyte membrane (Ω); A is the contact area between the electrode and the electrolyte membrane (cm^2^)

**Statistical analysis**

All statistical analyses were performed with Origin 2024 (Originlabs, USA). The peak fitting of the OH stretching band in in-situ infrared spectroscopy is achieved by using a Gaussian fitting algorithm. The current density is normalized according to the electrode area. In order to ensure the repeatability of data, we conducted three independent experiments on the Faraday efficiency and the yield of H_2_O_2_. In the body of the manuscript, the arithmetic means and standard deviation of all the samples are presented.


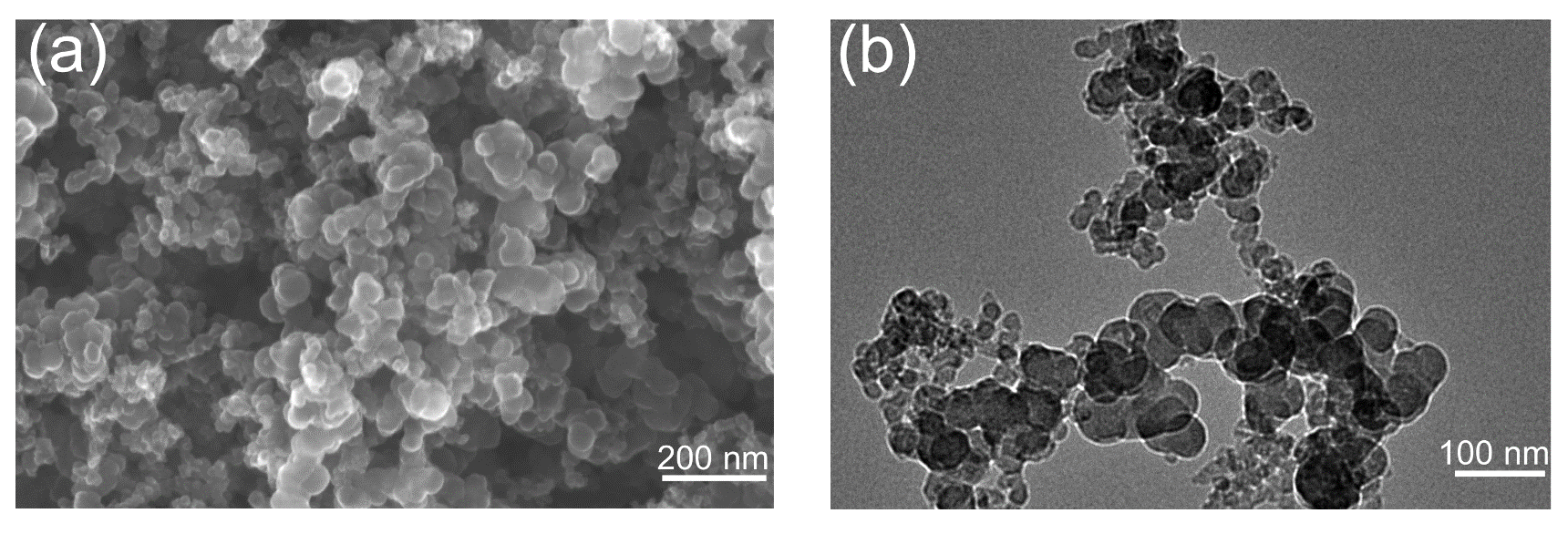


**Figure S1. Characterization of commercial carbon black (CB).** (**a**) SEM image of CB. (**b**) TEM image of CB.


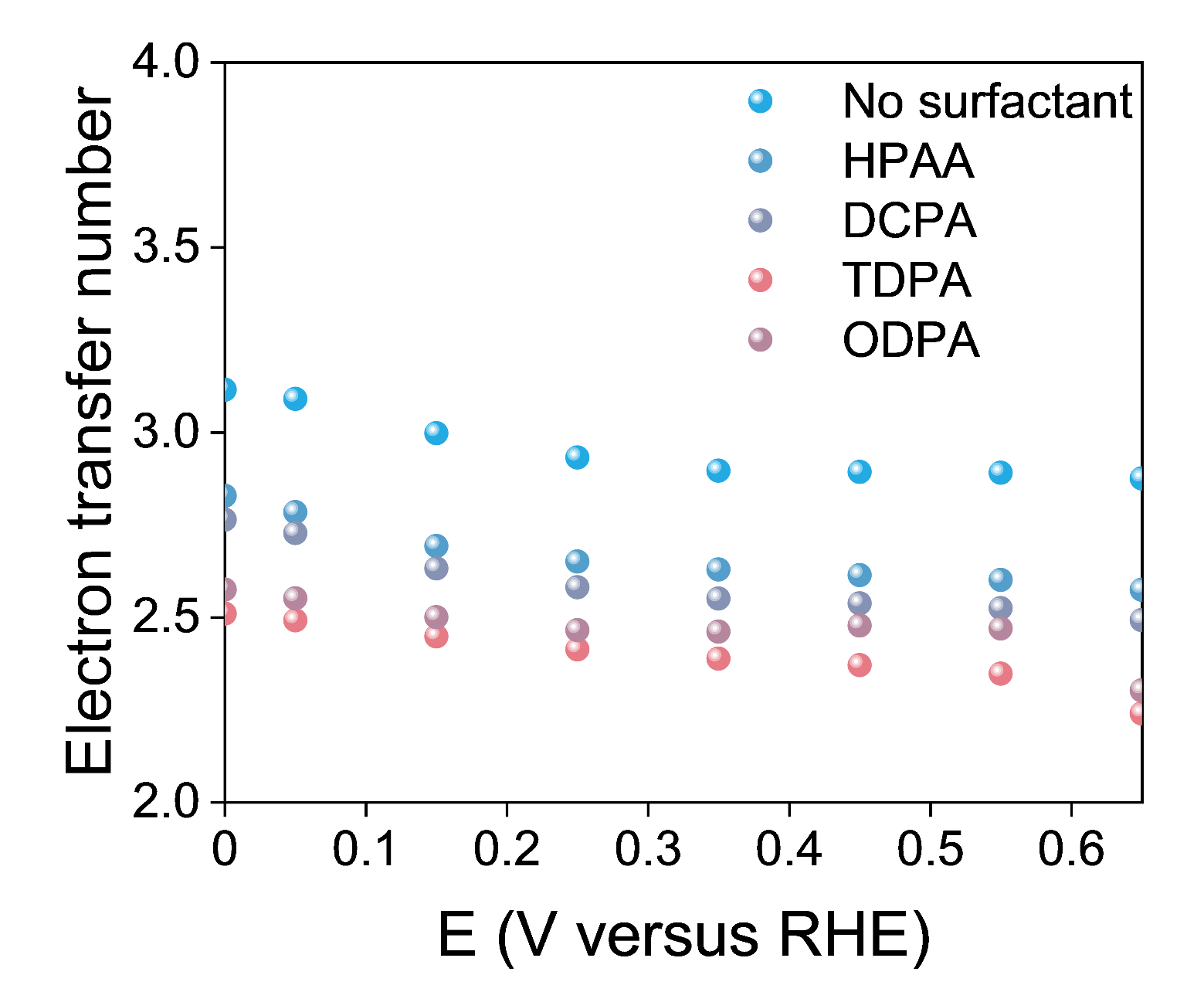


**Figure S2.** ORR electron transfer number of CB catalyst in different surfactant modified electrolytes from 0 to 0.65 V_RHE_.


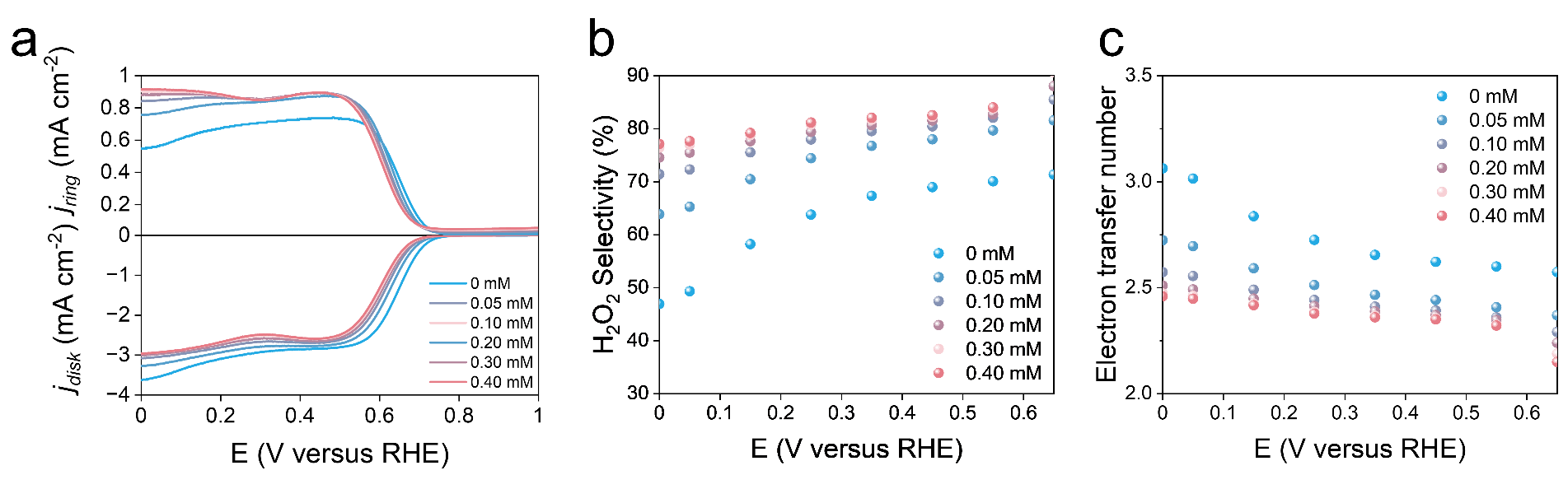


**Figure S3. ORR activity and selectivity of CB in electrolytes with different concentrations of TDPA.** (**a**) LSV curves of CB in O_2_-saturated 0.1 M KOH with different concentrations of TDPA (0−0.4 mM). (**b**) H_2_O_2_ selectivity in corresponding electrolytes. (**c**) Electron transfer number in corresponding electrolytes.


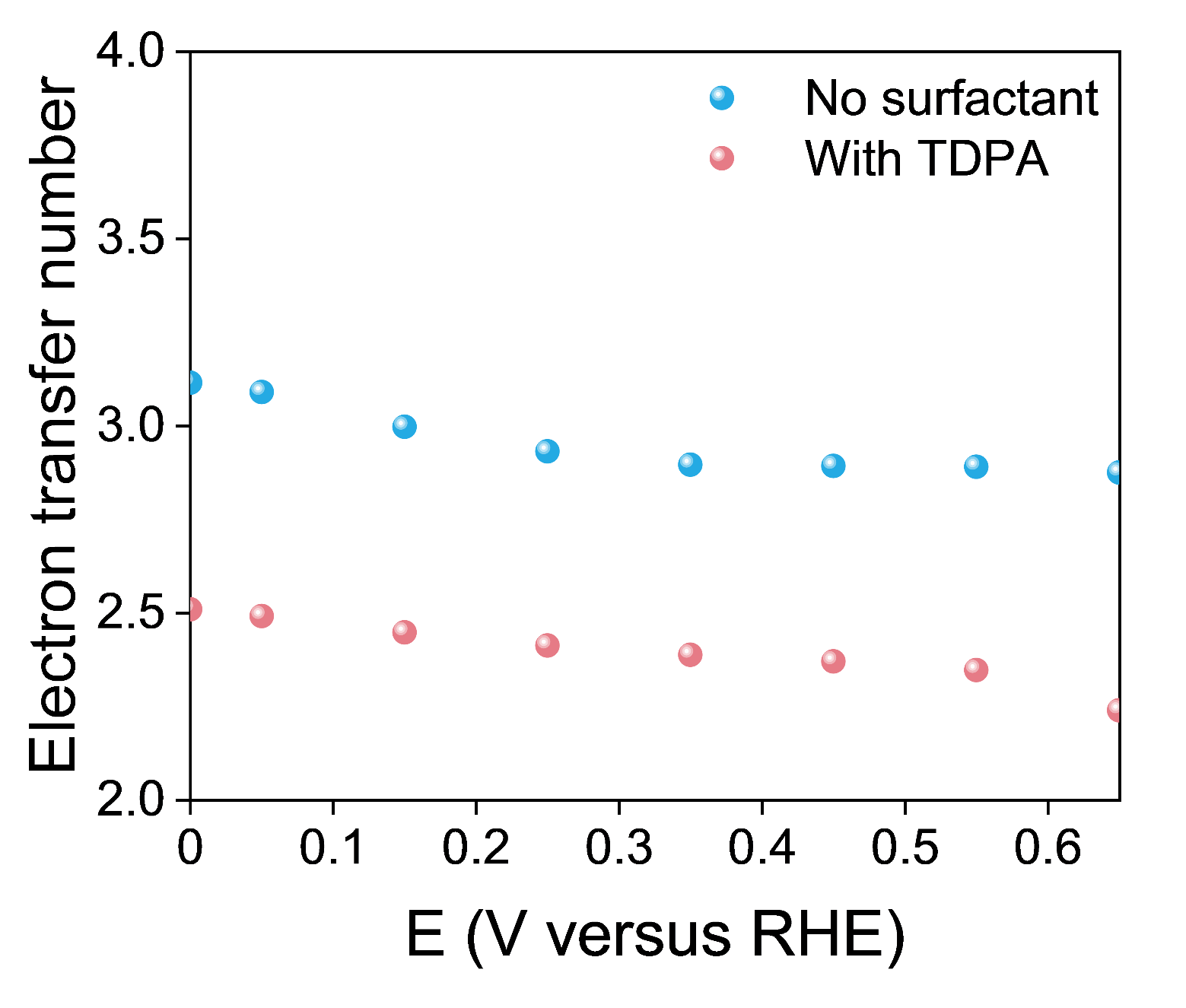


**Figure S4.** ORR electron transfer number of CB catalyst in 0.1 M KOH electrolyte with and without TDPA from 0 to 0.65 V_RHE_.


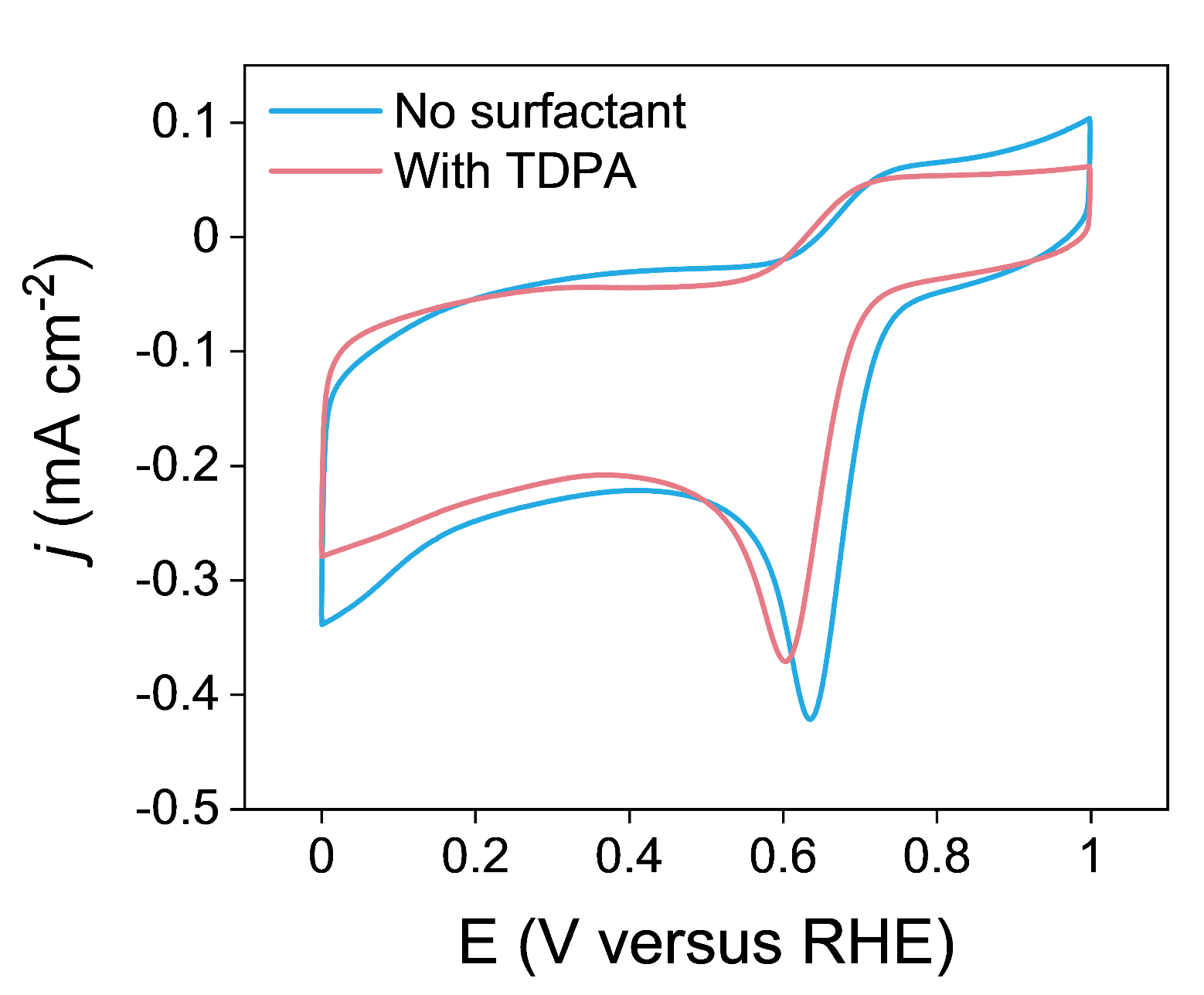


**Figure S5.** CV curves of CB catalyst in 0.1 M KOH electrolyte with and without TDPA from 0 to 1 V_RHE_.


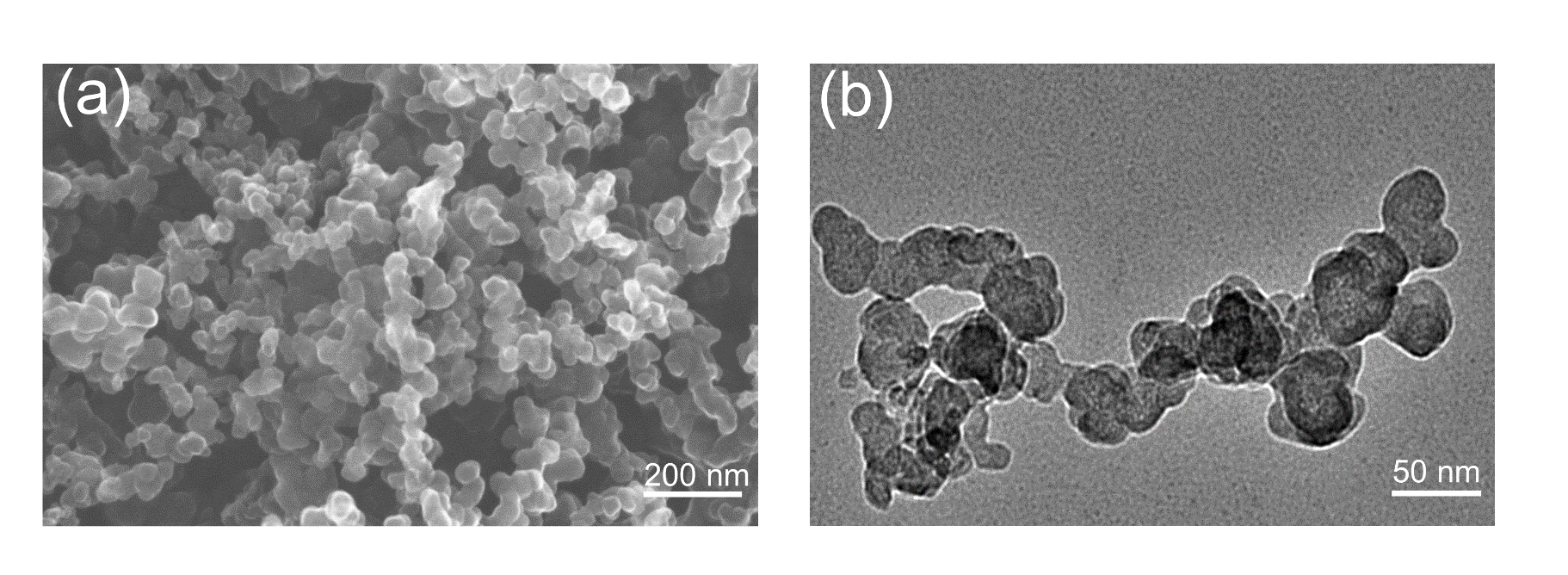


**Figure S6. Characterization of commercial Acetylene black (ACET).** (**a**) SEM image of ACET. (**b**) TEM image of ACET.


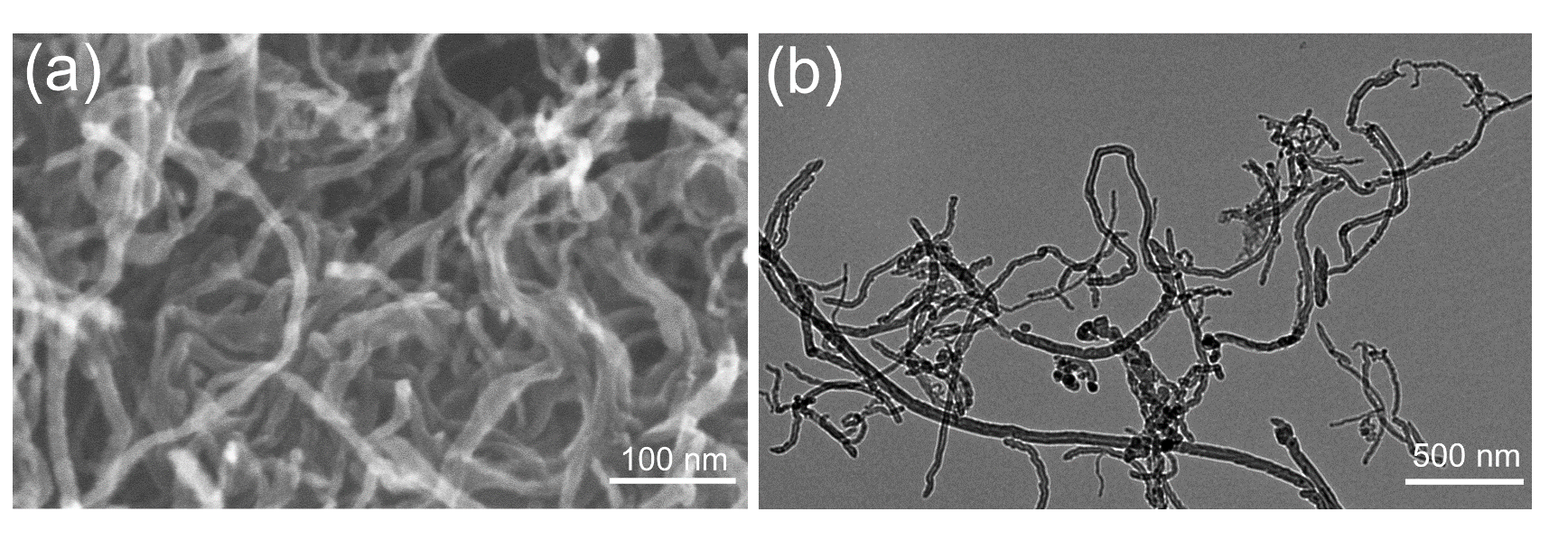


**Figure S7. Characterization of commercial Carbon nanotubes (CNT).** (**a**) SEM image of CNT. (**b**) TEM image of CNT.


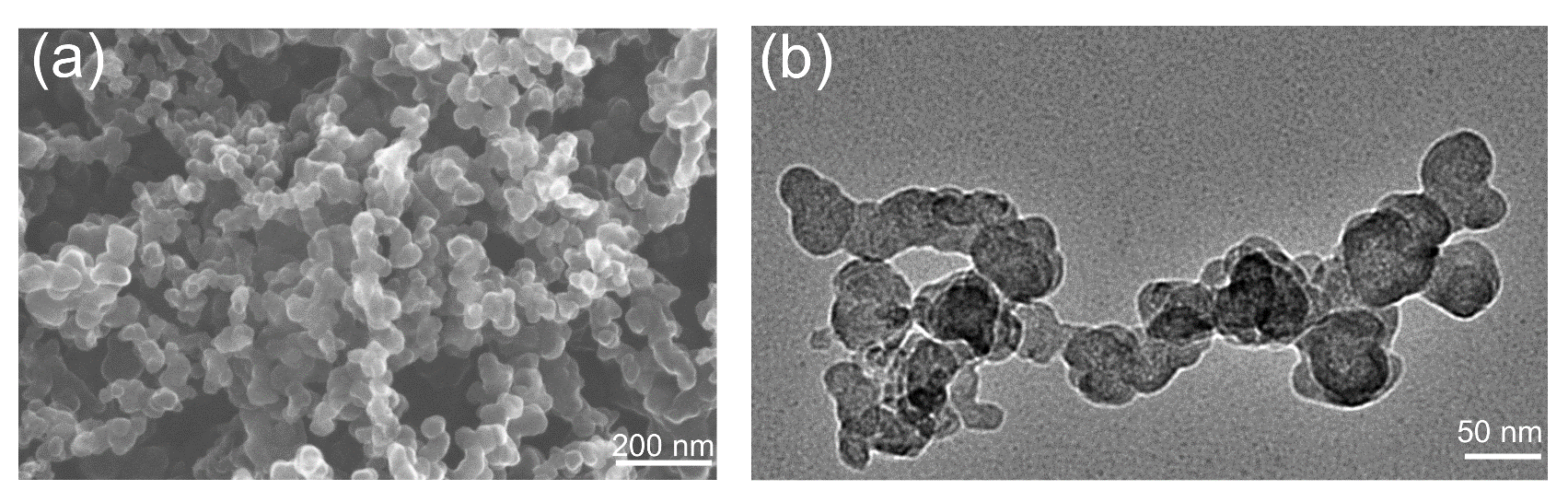


**Figure S8. Characterization of commercial Ketjen black (KB).** (**a**) SEM image of KB. (**b**) TEM image of KB.


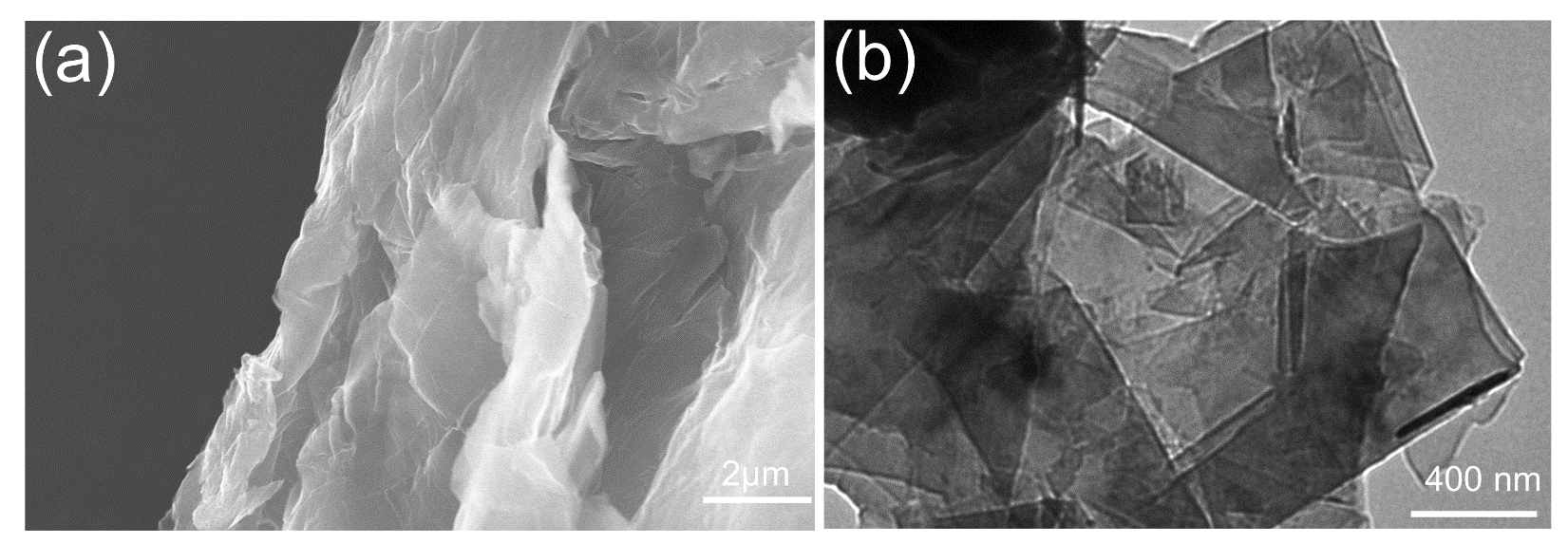


**Figure S9. Characterization of commercial reduced graphene oxide nanosheets (rGO).** (**a**) SEM image of rGO. (**b**) TEM image of rGO.


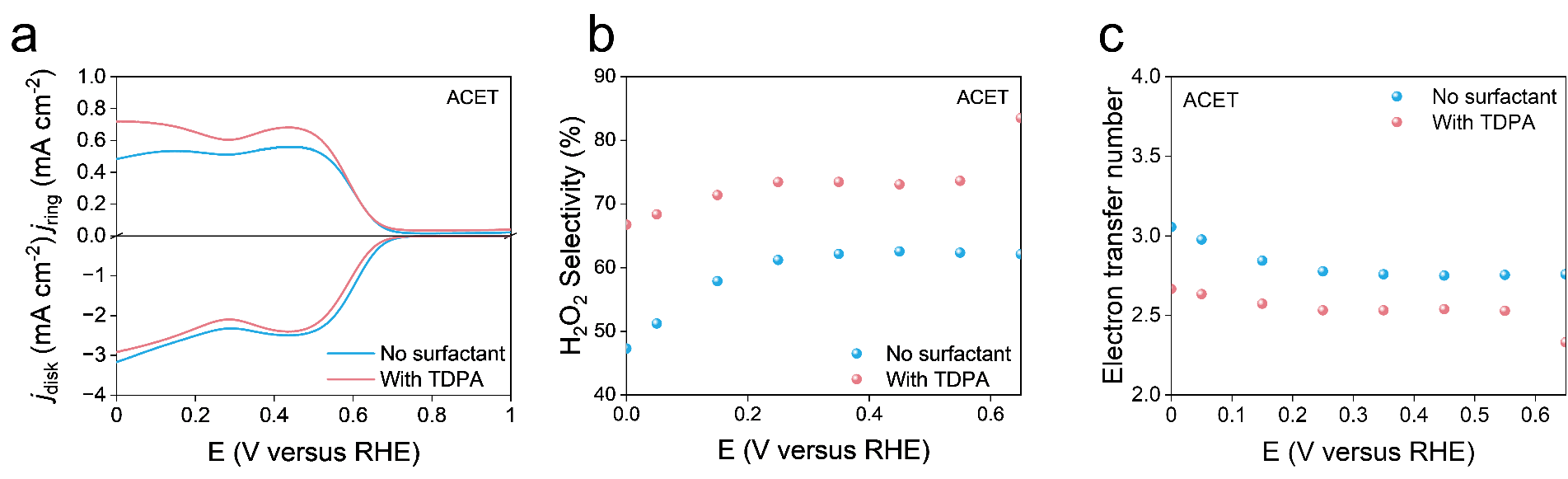


**Figure S10. ORR activity and selectivity of ACET in 0.1 M KOH electrolytes with and without TDPA.** (**a**) LSV curves of ACET in O_2_-saturated 0.1 M KOH with and without TDPA. (**b**) H_2_O_2_ selectivity in corresponding electrolytes. (**c**) Electron transfer number in corresponding electrolytes.


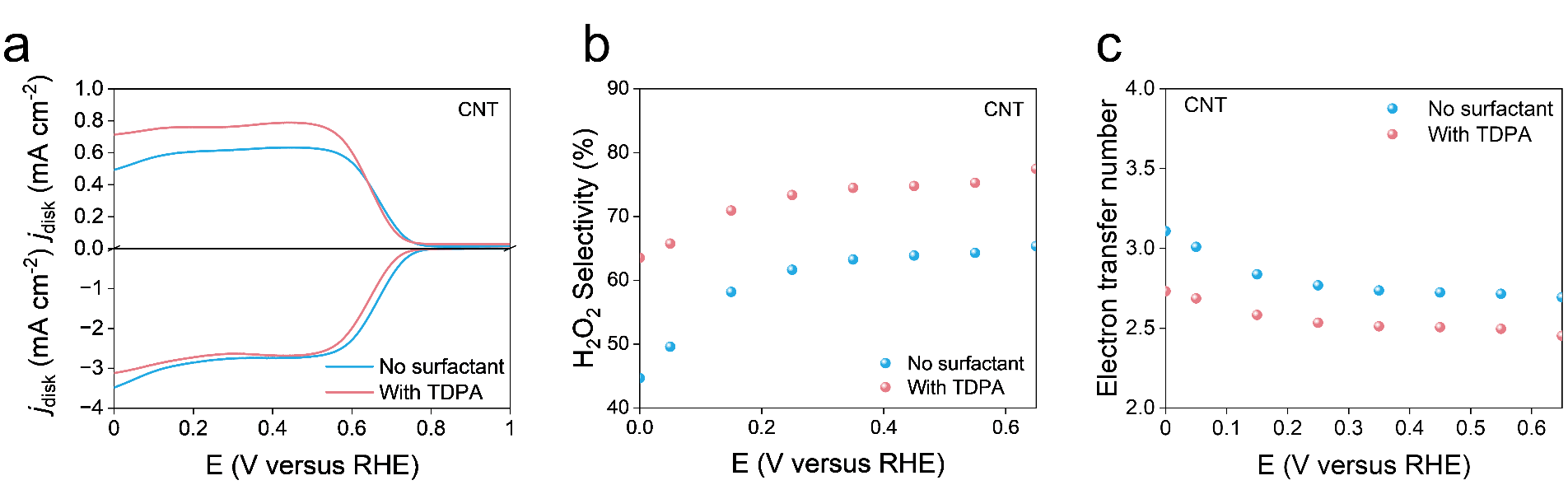


**Figure S11. ORR activity and selectivity of CNT in 0.1 M KOH electrolytes with and without TDPA.** (**a**) LSV curves of CNT in O_2_-saturated 0.1 M KOH with and without TDPA. (**b**) H_2_O_2_ selectivity in corresponding electrolytes. (**c**) Electron transfer number in corresponding electrolytes.


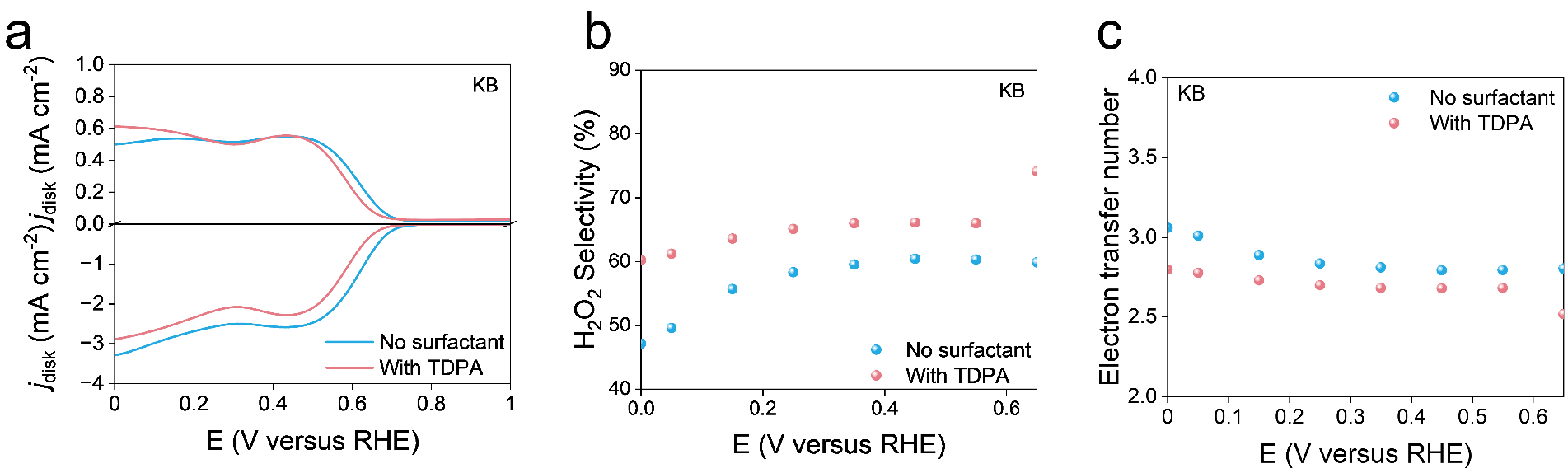


**Figure S12. ORR activity and selectivity of KB in 0.1 M KOH electrolytes with and without TDPA.** (**a**) LSV curves of KB in O_2_-saturated 0.1 M KOH with and without TDPA. (**b**) H_2_O_2_ selectivity in corresponding electrolytes. (**c**) Electron transfer number in corresponding electrolytes.


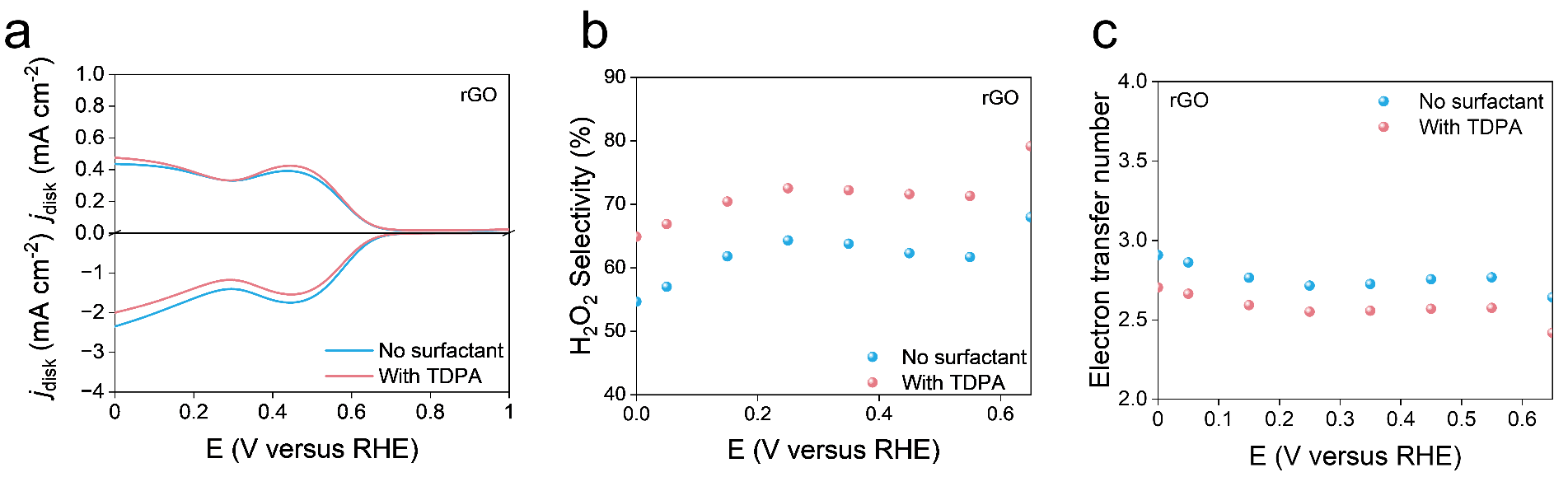


**Figure S13. ORR activity and selectivity of rGO in 0.1 M KOH electrolytes with and without TDPA.** (**a**) LSV curves of rGO in O_2_-saturated 0.1 M KOH with and without TDPA. (**b**) H_2_O_2_ selectivity in corresponding electrolytes. (**c**) Electron transfer number in corresponding electrolytes.


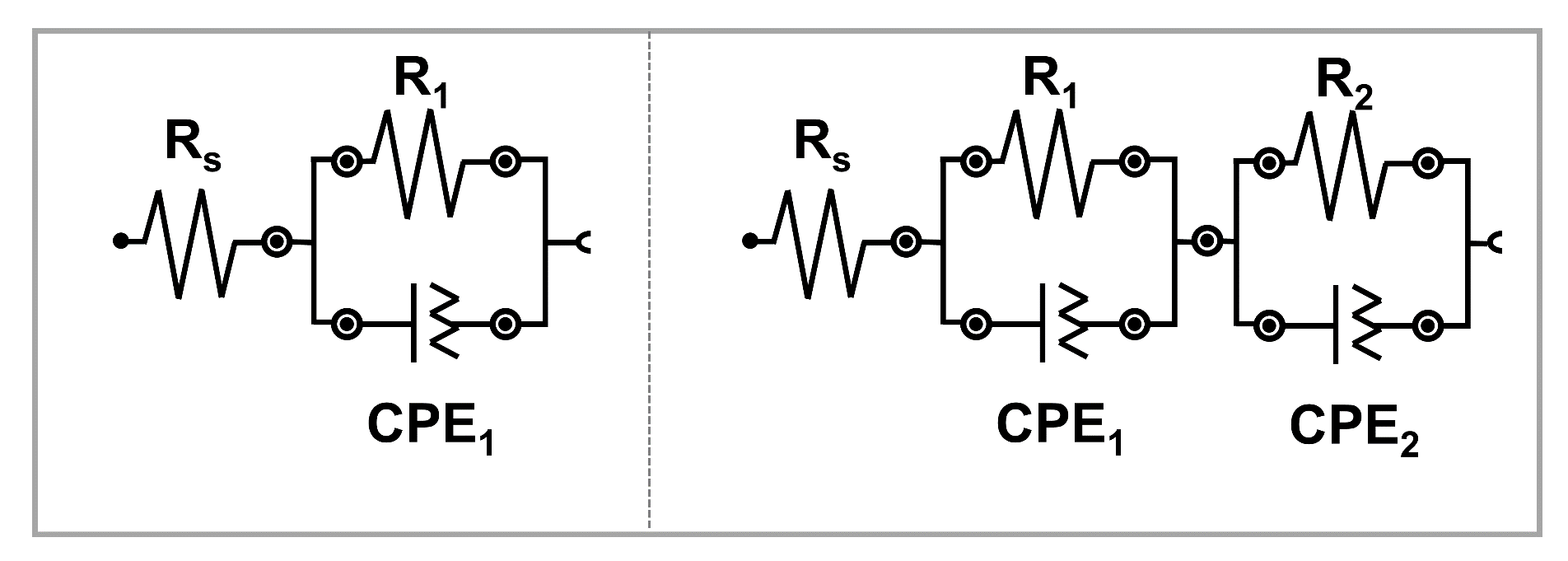


**Figure S14. The equivalent circuit diagrams.** The equivalent circuit diagram in KOH electrolyte with and without TDPA left (except from 0.65 V_RHE_ to 0.55 V_RHE_). The equivalent circuit diagram in KOH electrolyte right from 0.65 V_RHE_ to 0.55 V_RHE_.


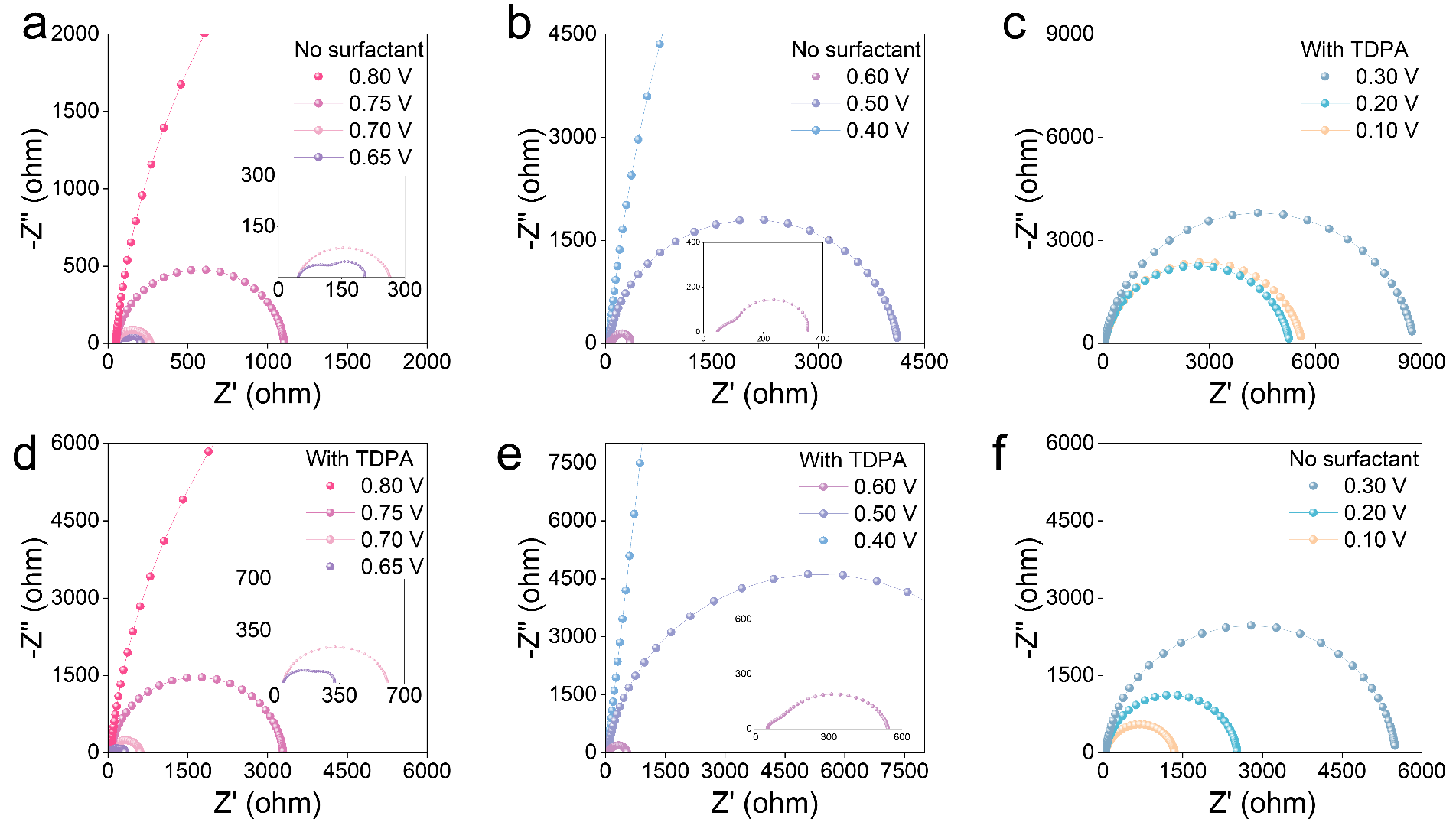


**Figure S15. Electrochemical impedance spectroscopy.** (**a**) Nyquist plots in KOH electrolyte from 0.8 to 0.65 V_RHE_. (**b**) Nyquist plots in KOH electrolyte from 0.6 to 0.4bV_RHE_. (**c**) Nyquist plots in KOH electrolyte from 0.3 to 0.1 V_RHE_. (**d**) Bode plots in KOH electrolyte from 0.8 to 0.65 V_RHE_. (**e**) Bode plots in KOH electrolyte from 0.6 to 0.4bV_RHE_. (**f**) Bode plots in KOH electrolyte from 0.3 to 0.1 V_RHE_.


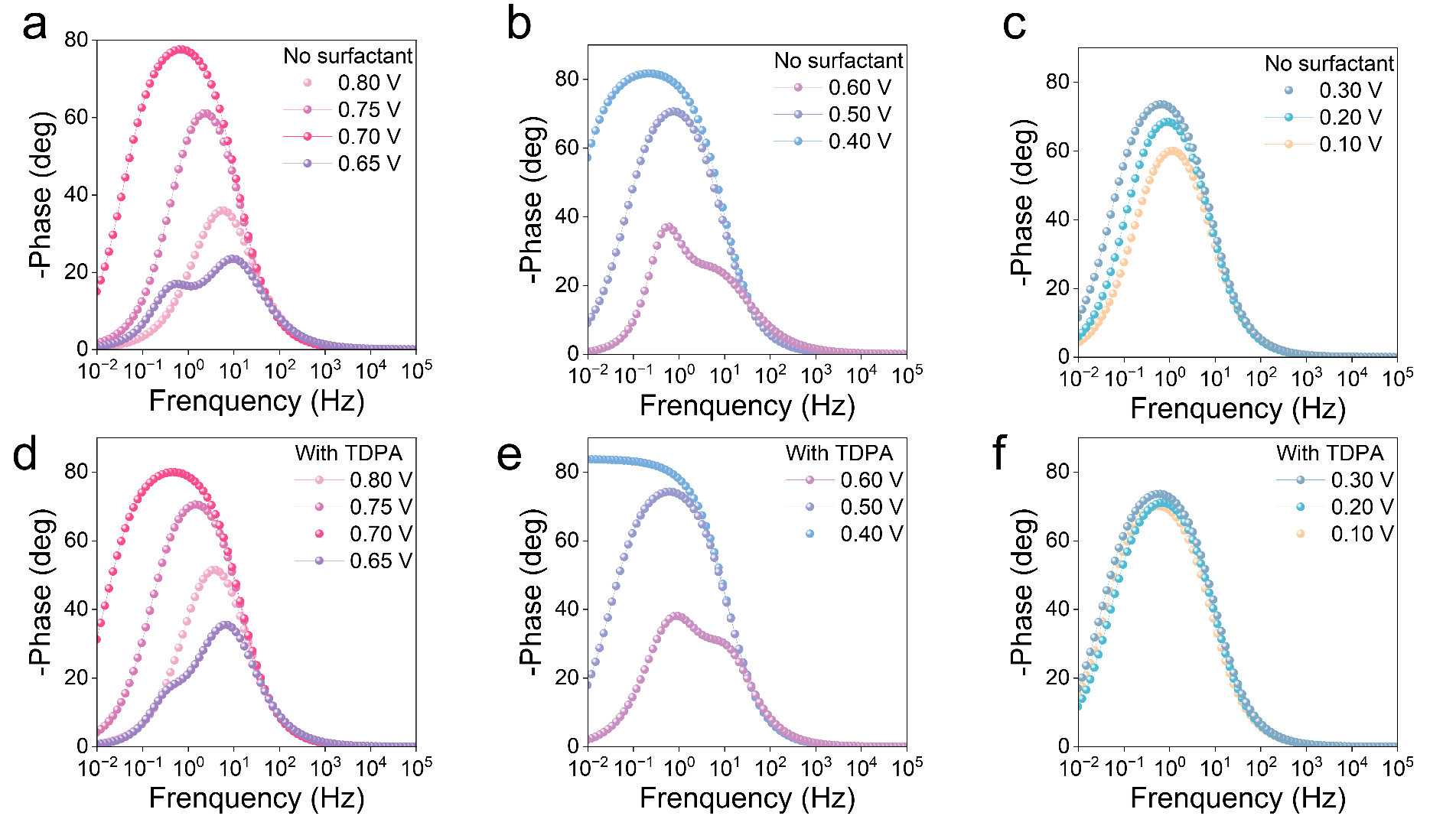


**Figure S16. Electrochemical impedance spectroscopy.** (**a**) Nyquist plots in KOH electrolyte with TDPA from 0.8 to 0.65 V_RHE_. (**b**) Nyquist plots in KOH electrolyte with TDPA from 0.6 to 0.4 V_RHE_. (**c**) Nyquist plots in KOH electrolyte with TDPA from 0.3 to 0.1 V_RHE_. (**d**) Bode plots in KOH electrolyte with TDPA from 0.8 to 0.65 V_RHE_. (e) Bode plots in KOH electrolyte with TDPA from 0.6 to 0.4bV_RHE_. (**f**) Bode plots in KOH electrolyte with TDPA from 0.3 to 0.1 V_RHE_.


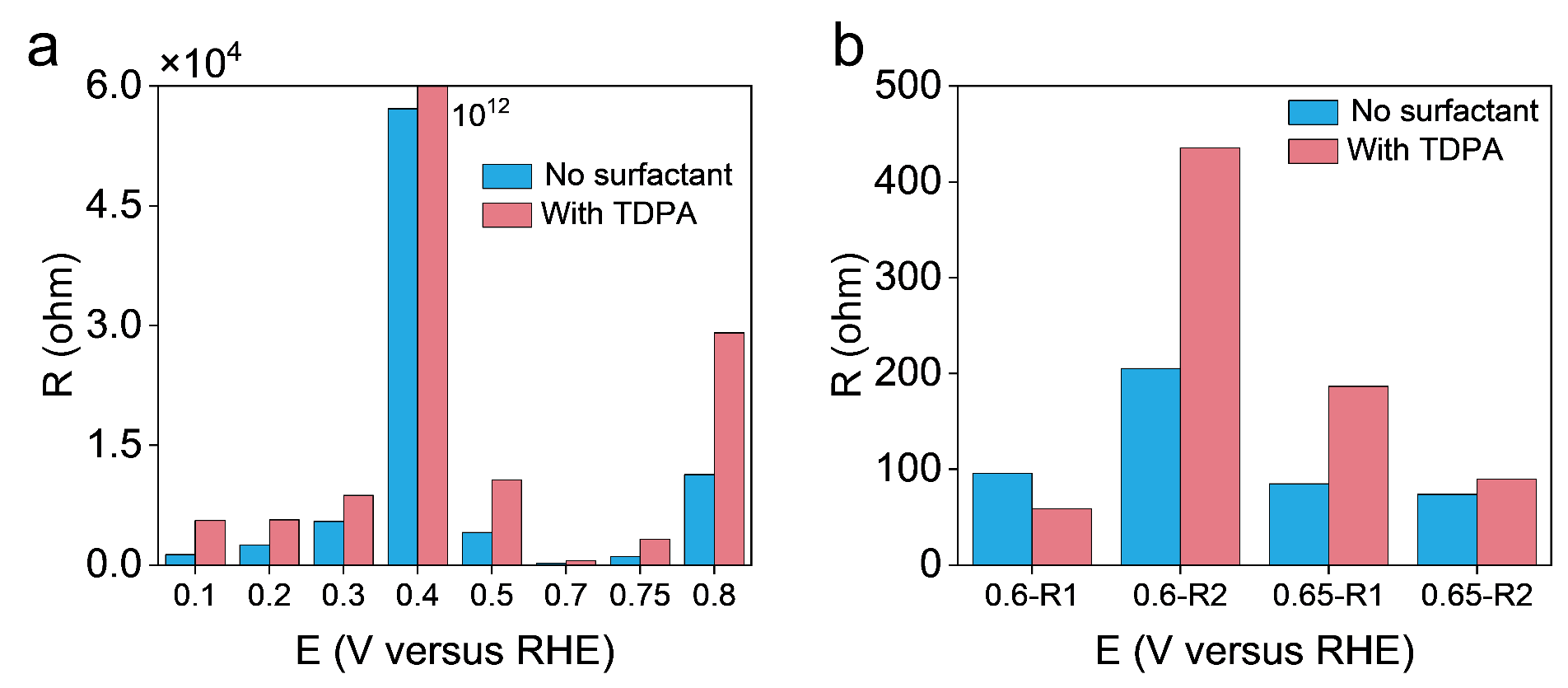


**Figure S17. Electrochemical impedance spectroscopy.** (**a**) Resistance in KOH electrolyte with and without TDPA from 0.8 to 0.1 V_RHE_. (**b**) Resistance in KOH electrolyte with and without TDPA at 0.6 and 0.65 V_RHE_. The Resistance (R) is R_1_ and R_2_.


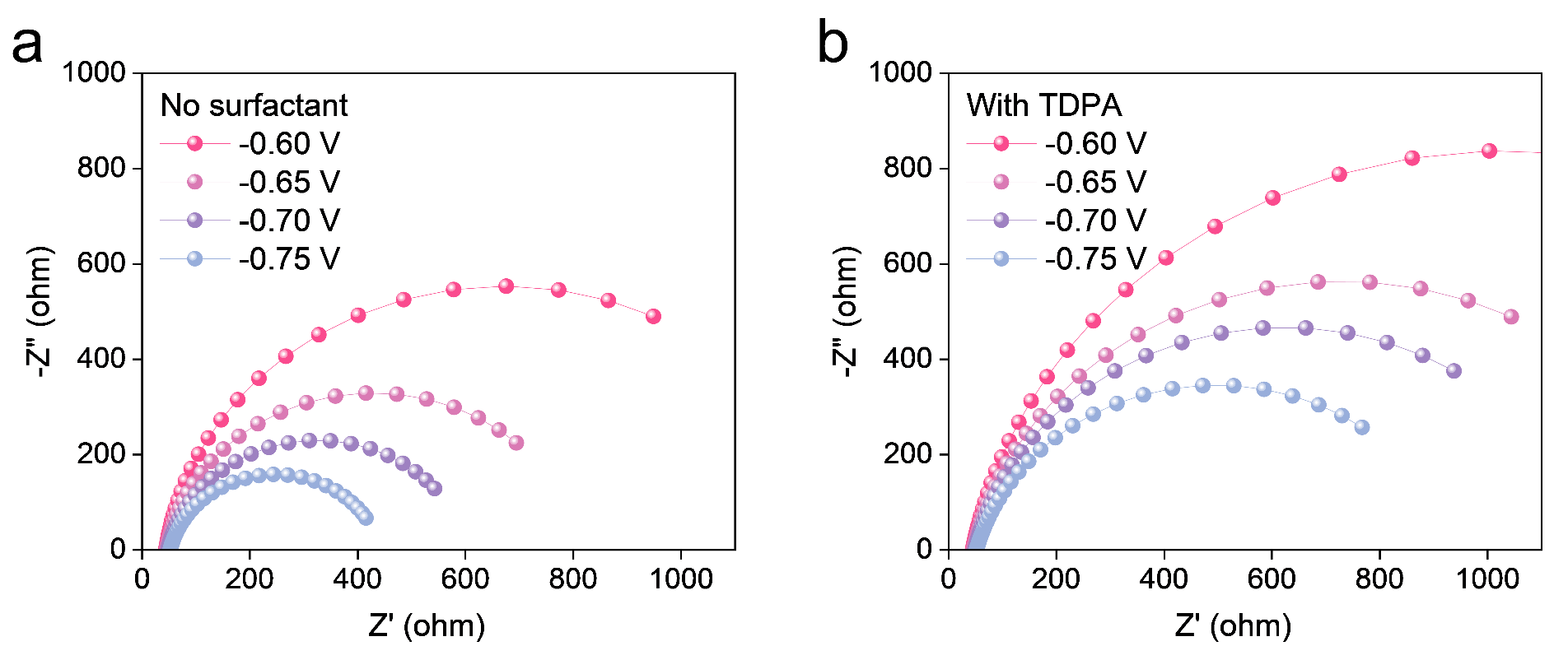


**Figure S18. Electrochemical impedance spectroscopy.** (**a**) Nyquist plots in KOH electrolyte from −0.6 to −0.75 V_RHE_. (**b**) Nyquist plots in KOH electrolyte with TDPA from −0.6 to −0.75 V_RHE_.


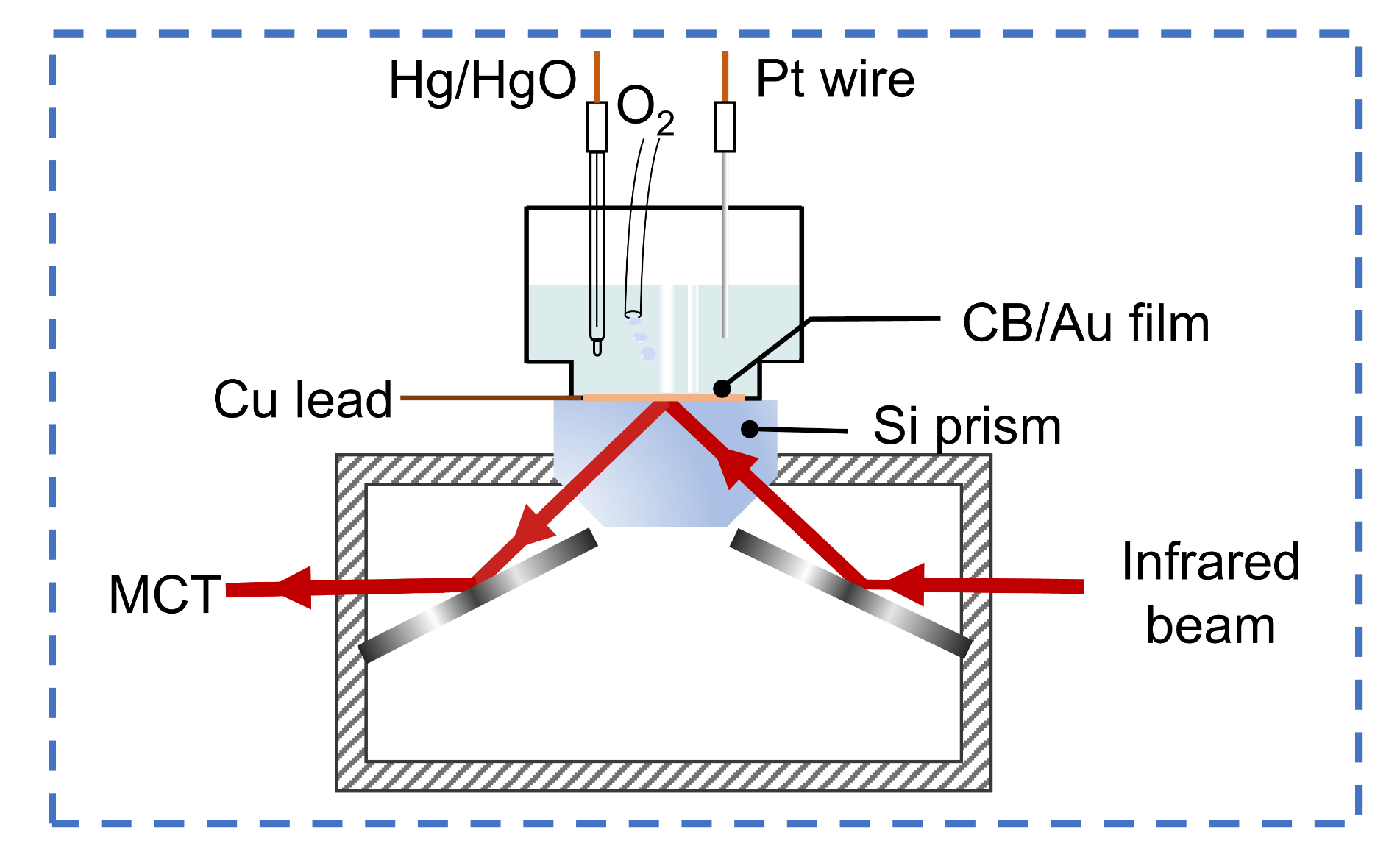


**Figure S19.** Schematic diagram of ATR-SEIRAS.


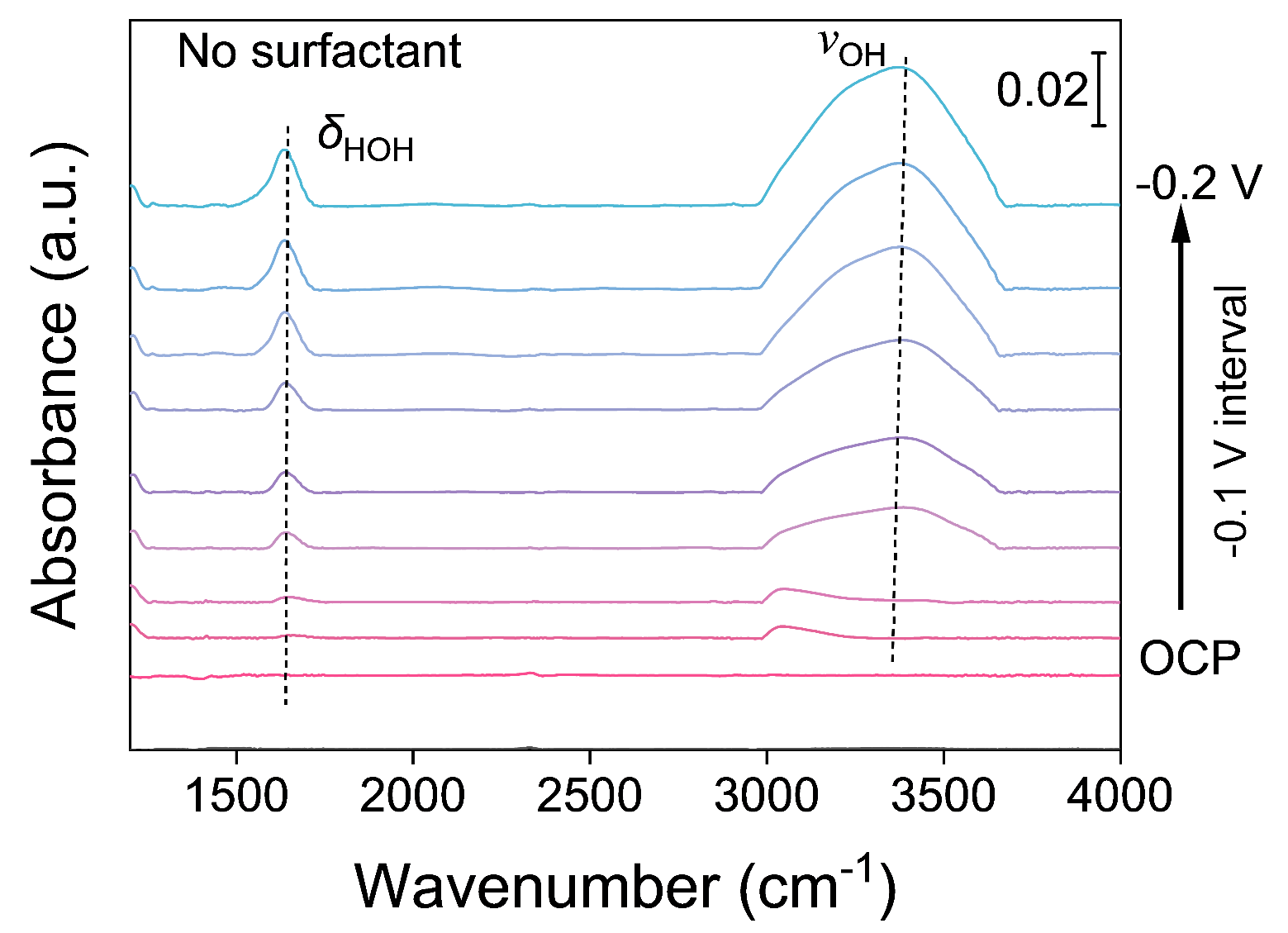


**Figure S20.** In situ ATR-SEIRAS spectra of the KOH electrolyte in the range of 1200−3000 cm^−1^ at varied potentials (from 0.8 to −0.3 V_RHE_).


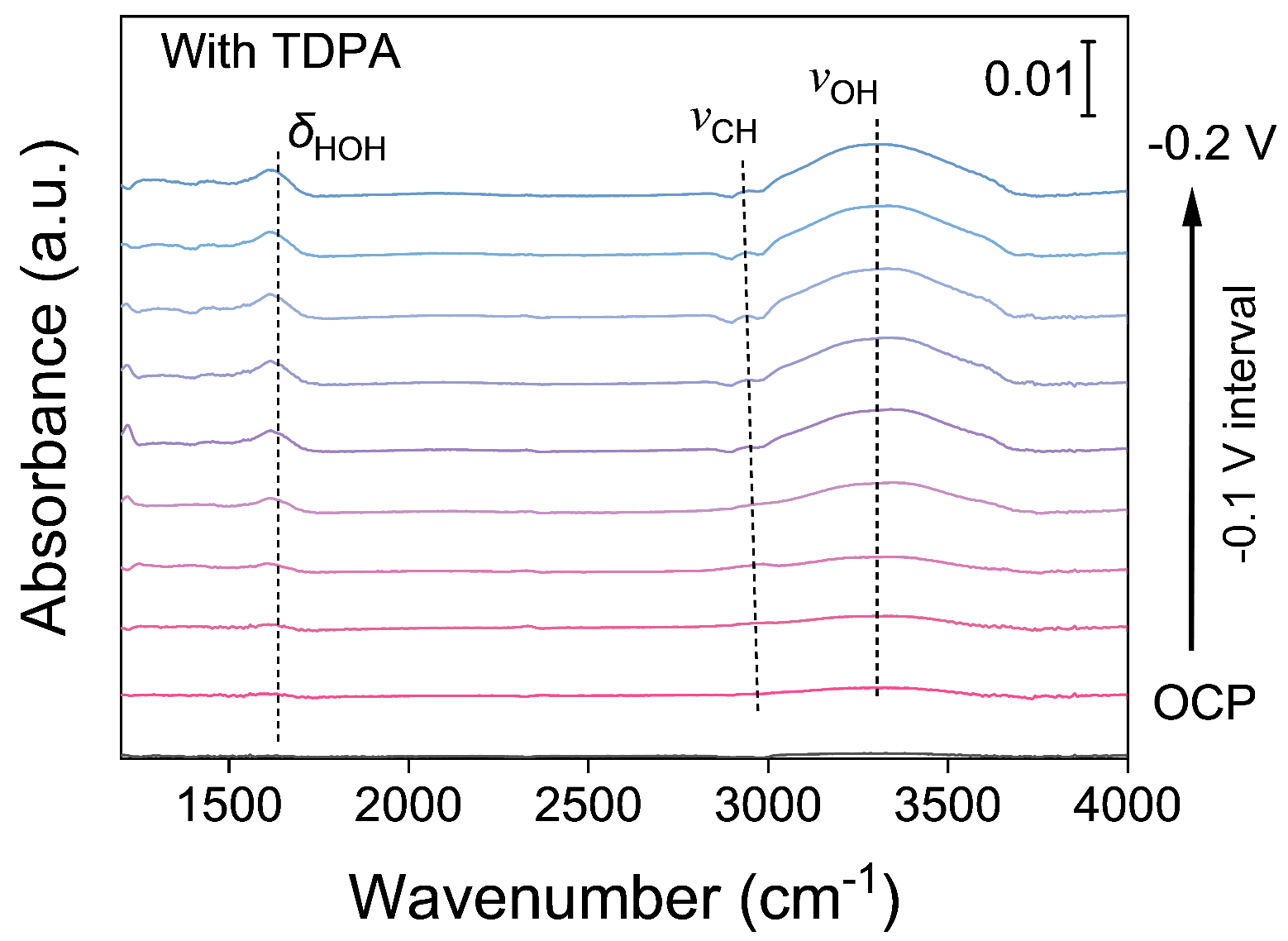


**Figure S21.** In situ ATR-SEIRAS spectra of the KOH electrolyte in the range of 1200−3000 cm^−1^ at varied potentials (from 0.8 to −0.3 V_RHE_).


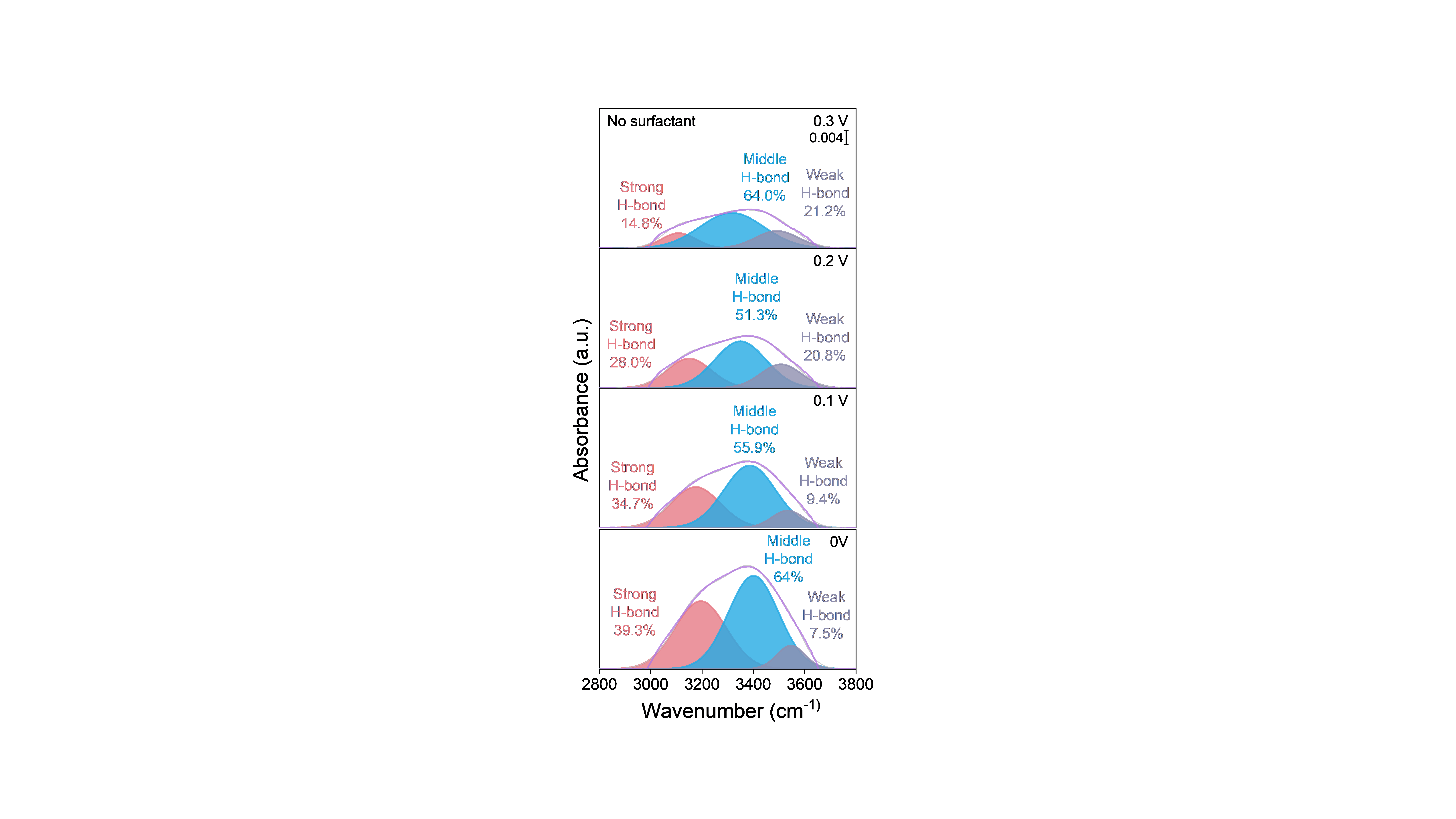


**Figure S22.** Intensity of three different types of *ν*-OH of water in KOH electrolyte from 0.3 to −0.2 V_RHE_. The peaks were split using a Gaussian simulation.


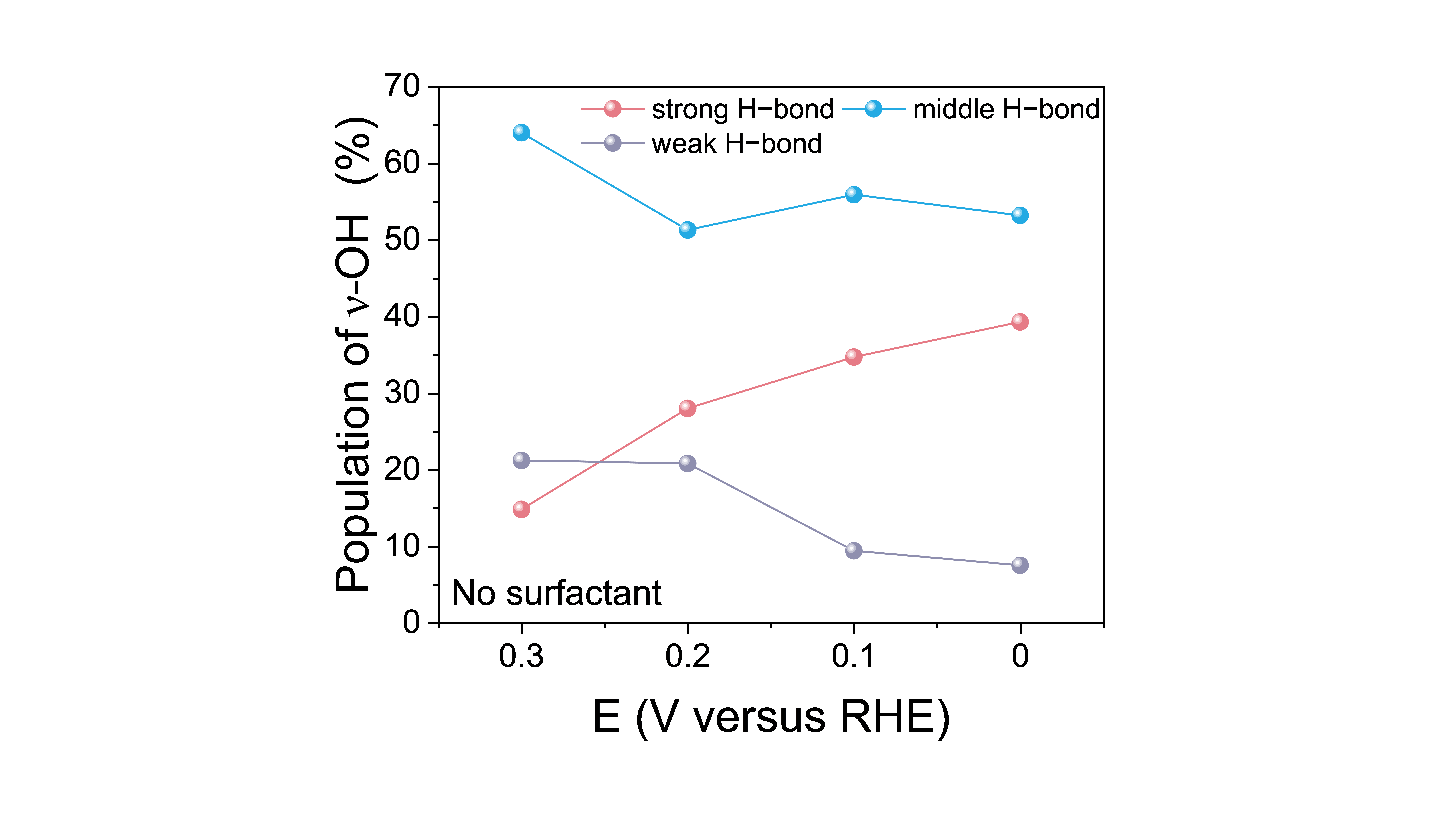


**Figure S23.** Trend chart of relative intensity about three O-H stretching modes of interfacial water in KOH electrolyte in the range of 2800−3800 cm^−1^.


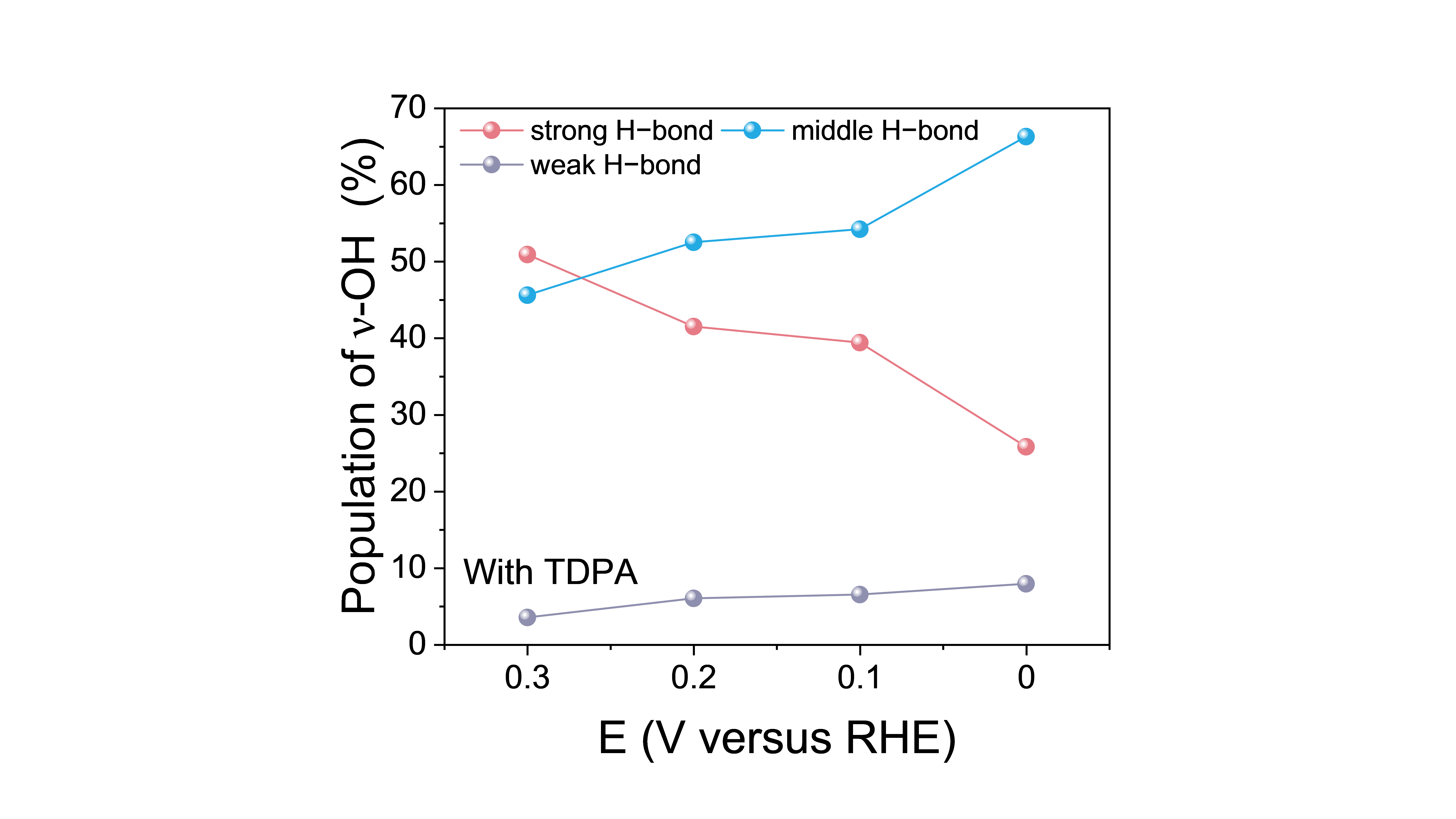


**Figure S24.** Trend chart of relative intensity about three O-H stretching modes of interfacial water in KOH electrolyte with TDPA in the range of 2800−3800 cm^−1^.


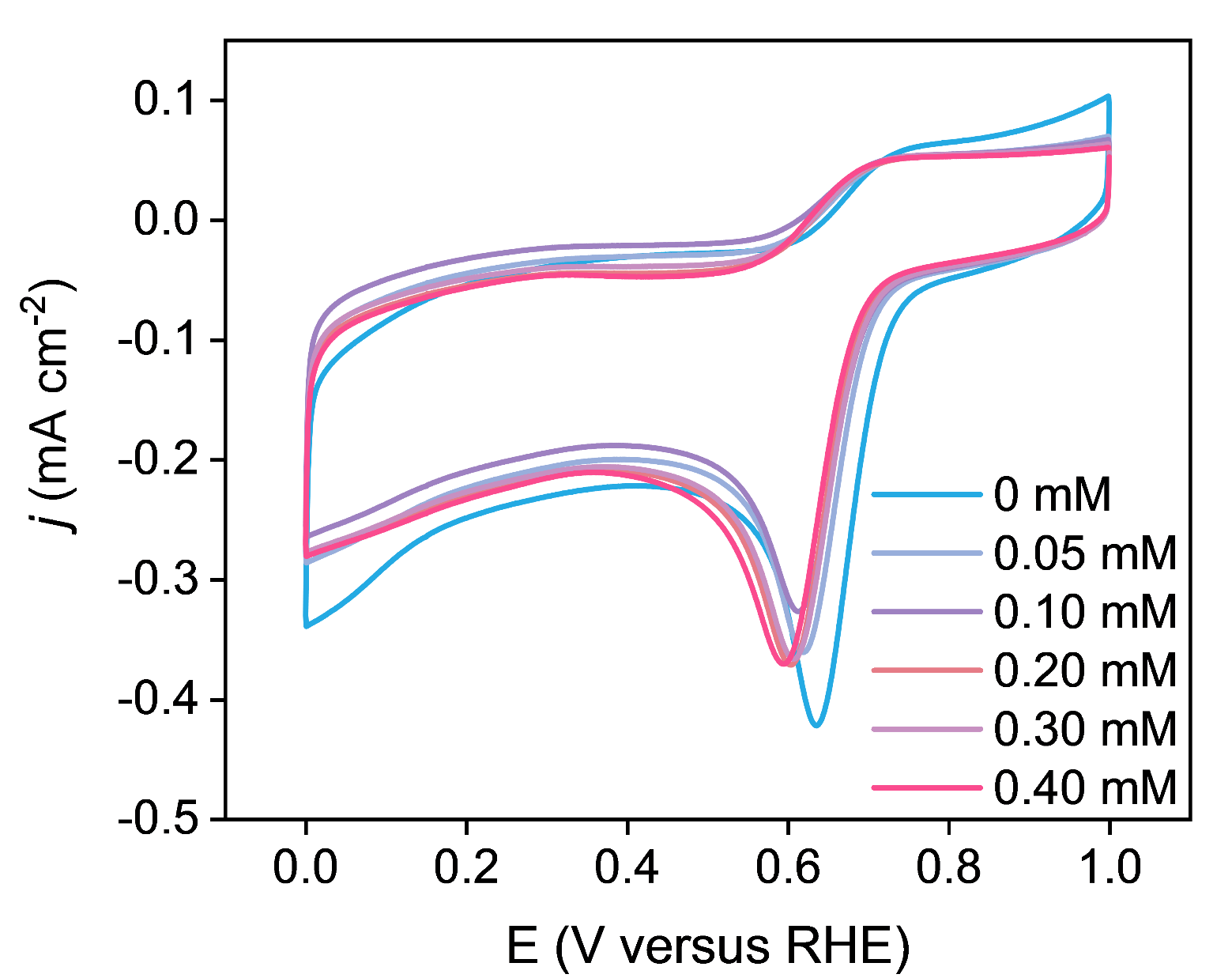


**Figure S25.** CV curves of CB catalyst in 0.1 M KOH electrolyte with different concentration of TDPA from 0 to 1 V_RHE_.


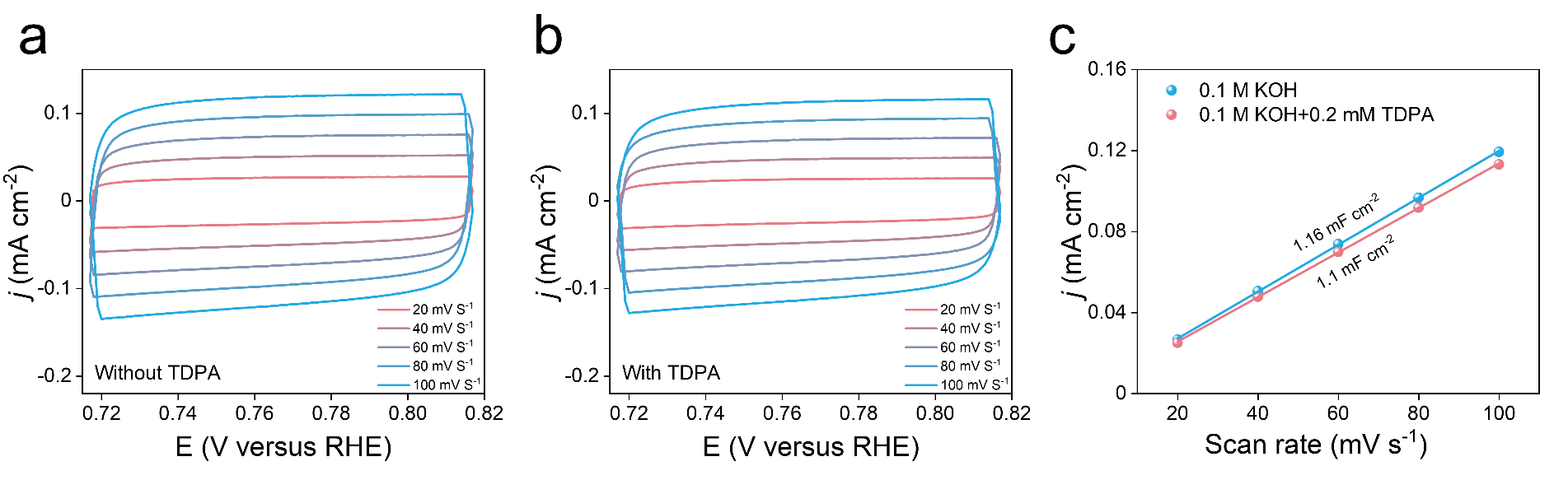


**Figure S26.** **Electrochemical active area.** (**a**) CV curves in double layer region at scan rates of 20, 40, 60, 80, 100 mV s^−1^ in N_2_-saturated 0.1 M KOH. (**b**) Cyclic voltammograms in double layer region at scan rates of 20, 40, 60, 80, 100 mV s^−1^ in N_2_-saturated 0.1 M KOH with TDPA. (**c**) Capacitance current densities measured at 0.765 V_RHE_ as a function of scan rate.


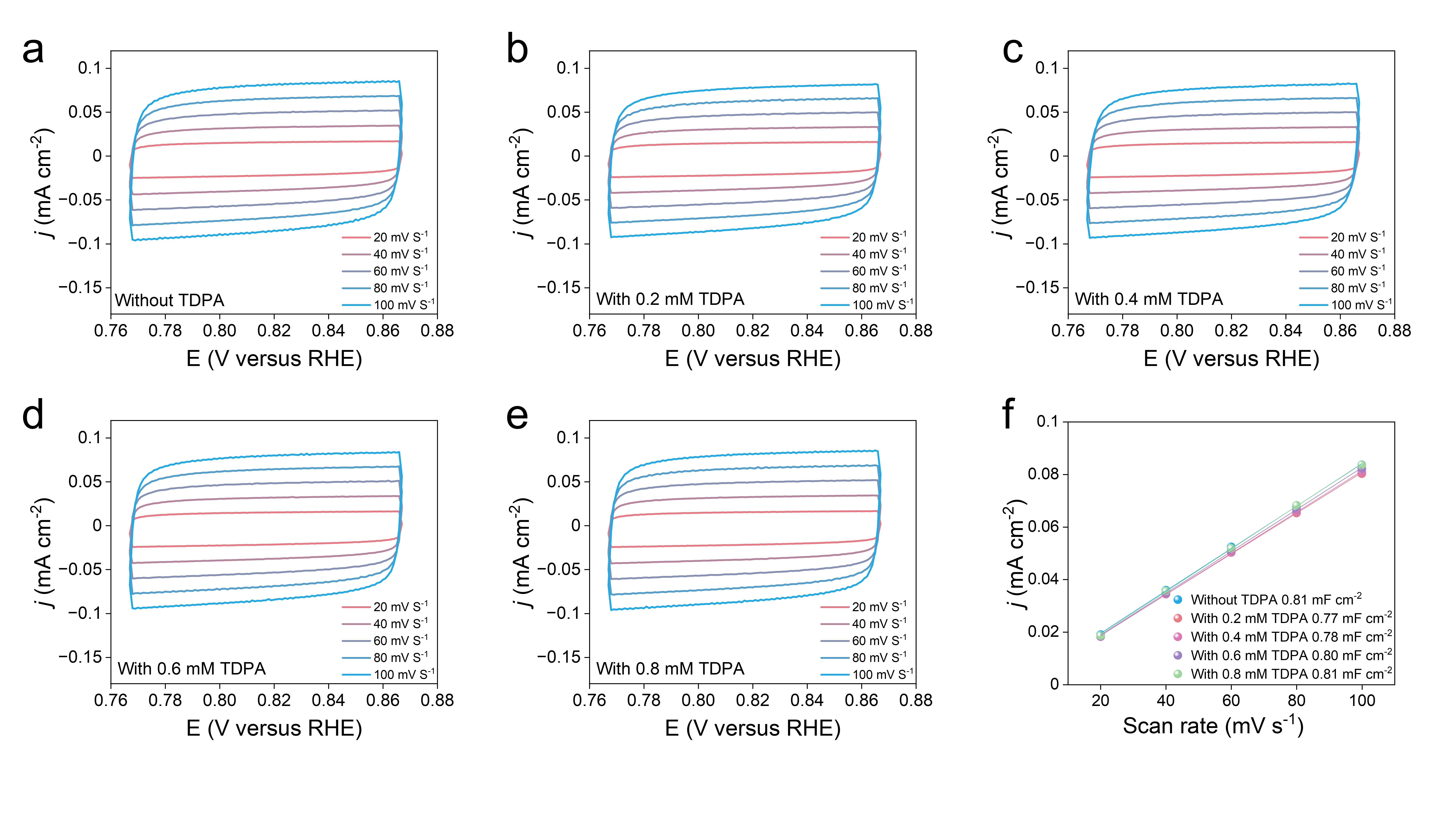


**Figure S27. Electrochemical active area.** (**a**), (**b**), (**c**), (**d**), and (**e**) CV curves in double layer region at scan rates of 20, 40, 60, 80, 100 mV s^−1^ in N_2_-saturated 0.1 M KOH without TDPA and with 0.2 mM, 0.4 mM, 0.6 mM, 0.8 mM TDPA. (**f**) Capacitance current densities measured at 0.817 V_RHE_ as a function of scan rate.


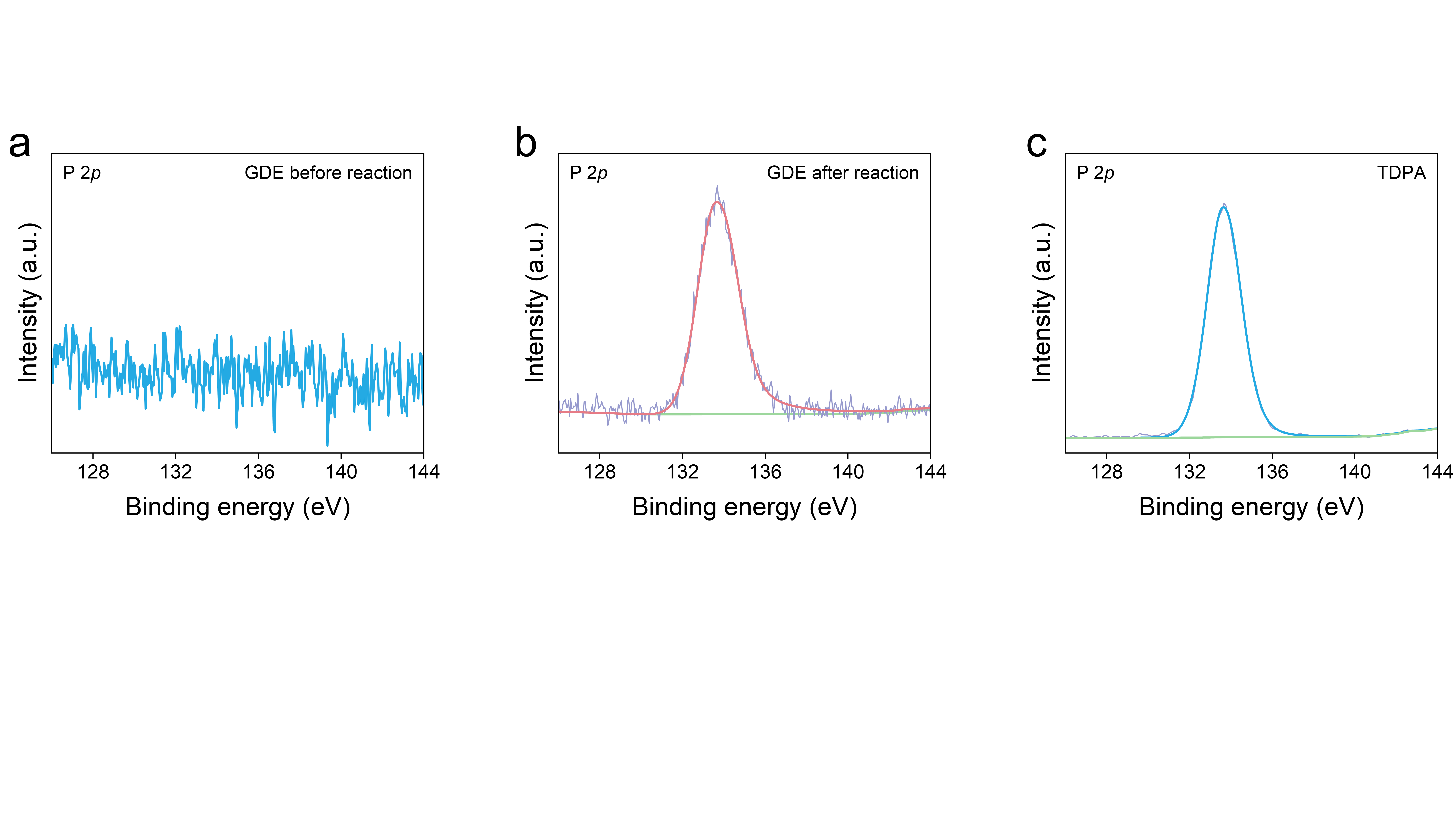


**Figure S28.** (**a**) XPS spectrum of P on GDE before the reaction in flow cell. (**b**) XPS spectrum of P on GDE after the reaction in flow cell. (**c**) XPS spectrum of P on TDPA.
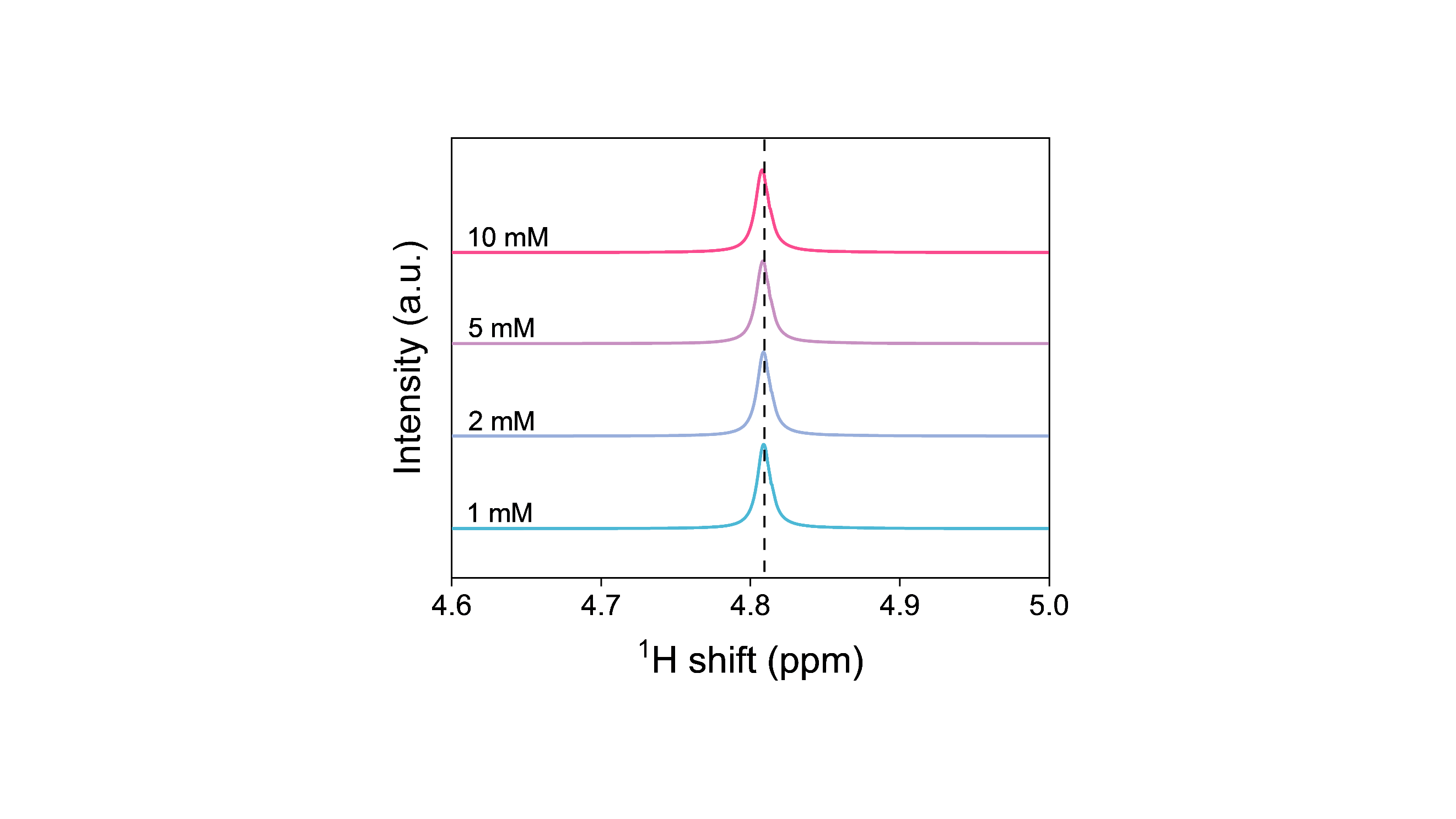


**Figure S29.** ^1^H NMR spectra of the KOH electrolytes with different concentrations of TDPA.


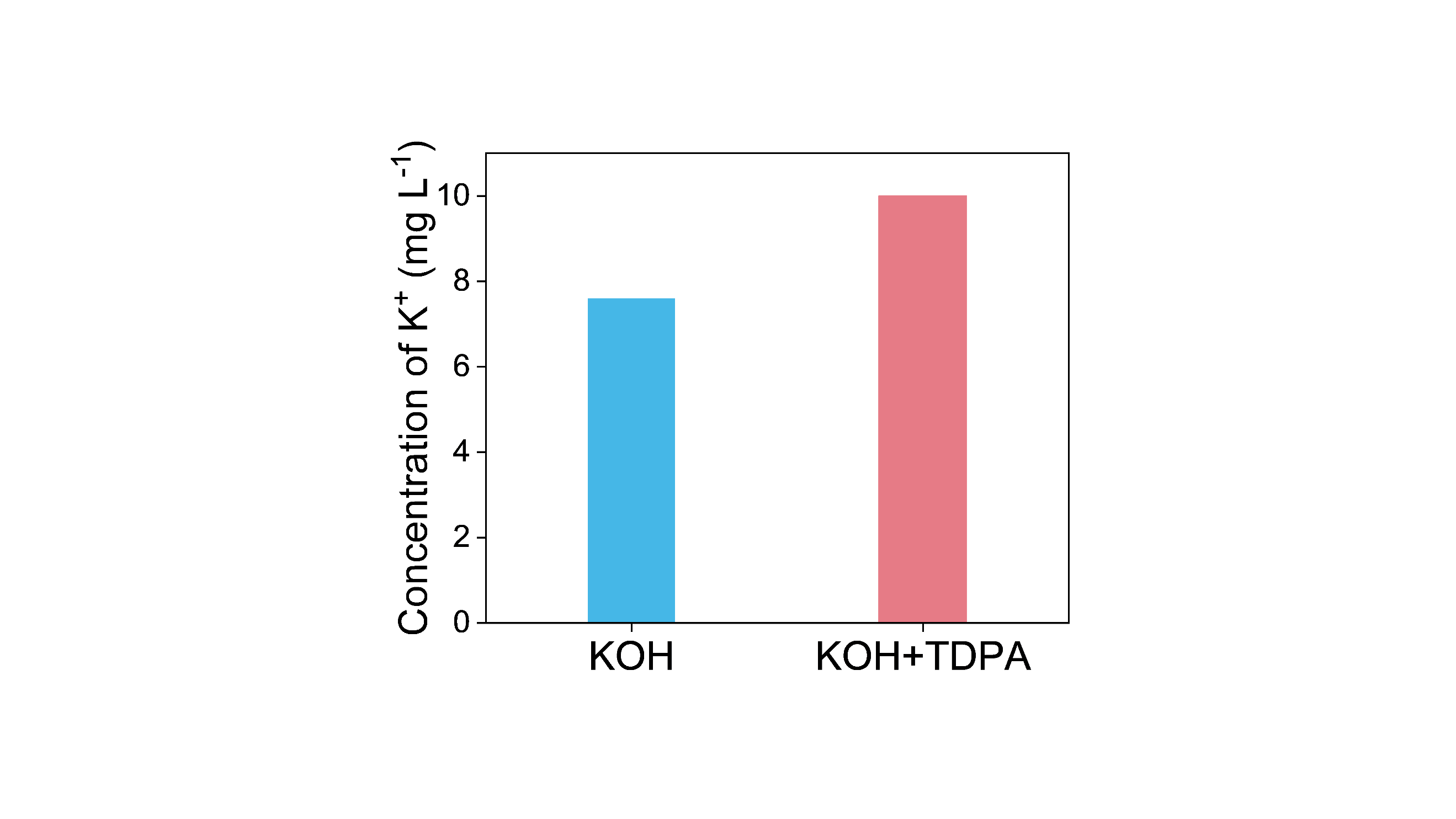


**Figure S30.** Absorbed K^+^ concentration in KOH electrolytes with and without TDPA.


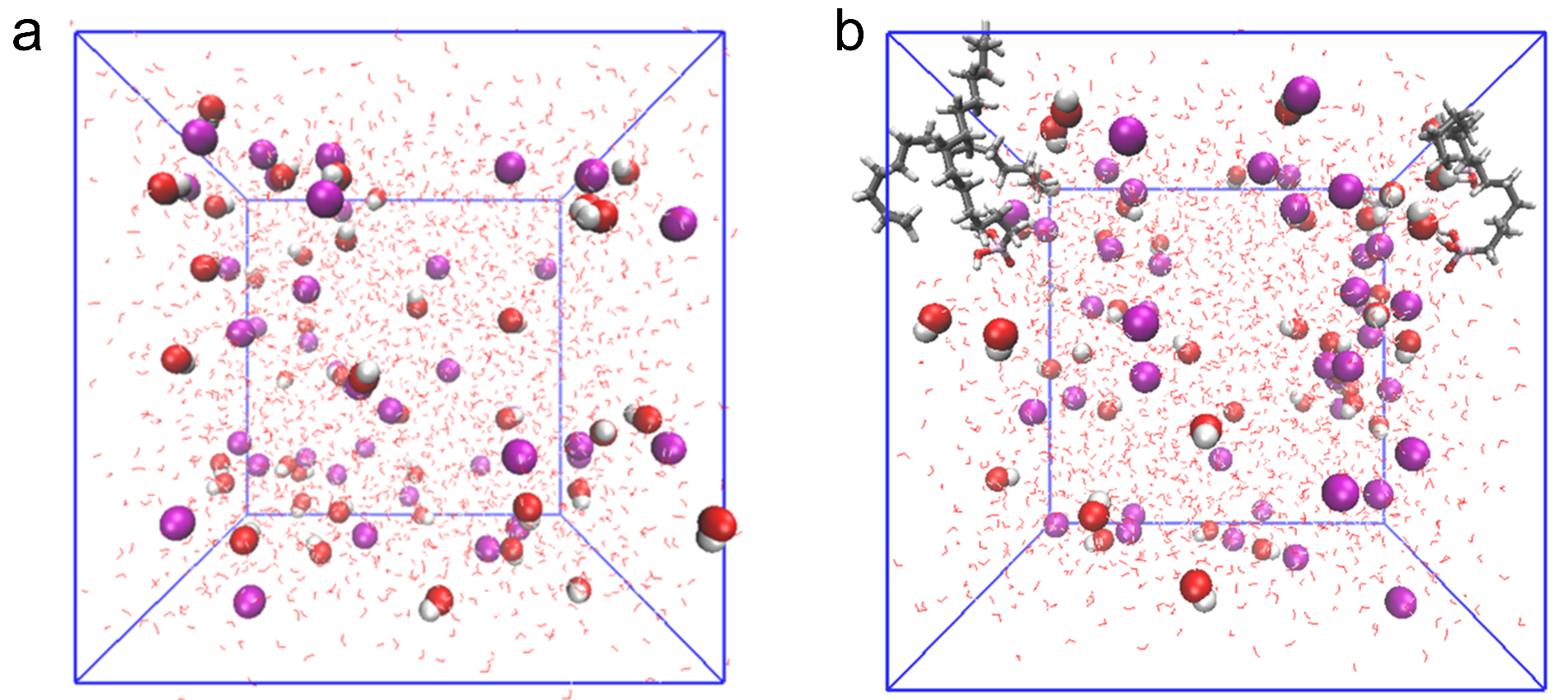


**Figure S31.** (**a**) 3D snapshot of the KOH electrolyte from MD simulations. (**b**) 3D snapshot of the KOH electrolyte with TDPA from MD simulations.


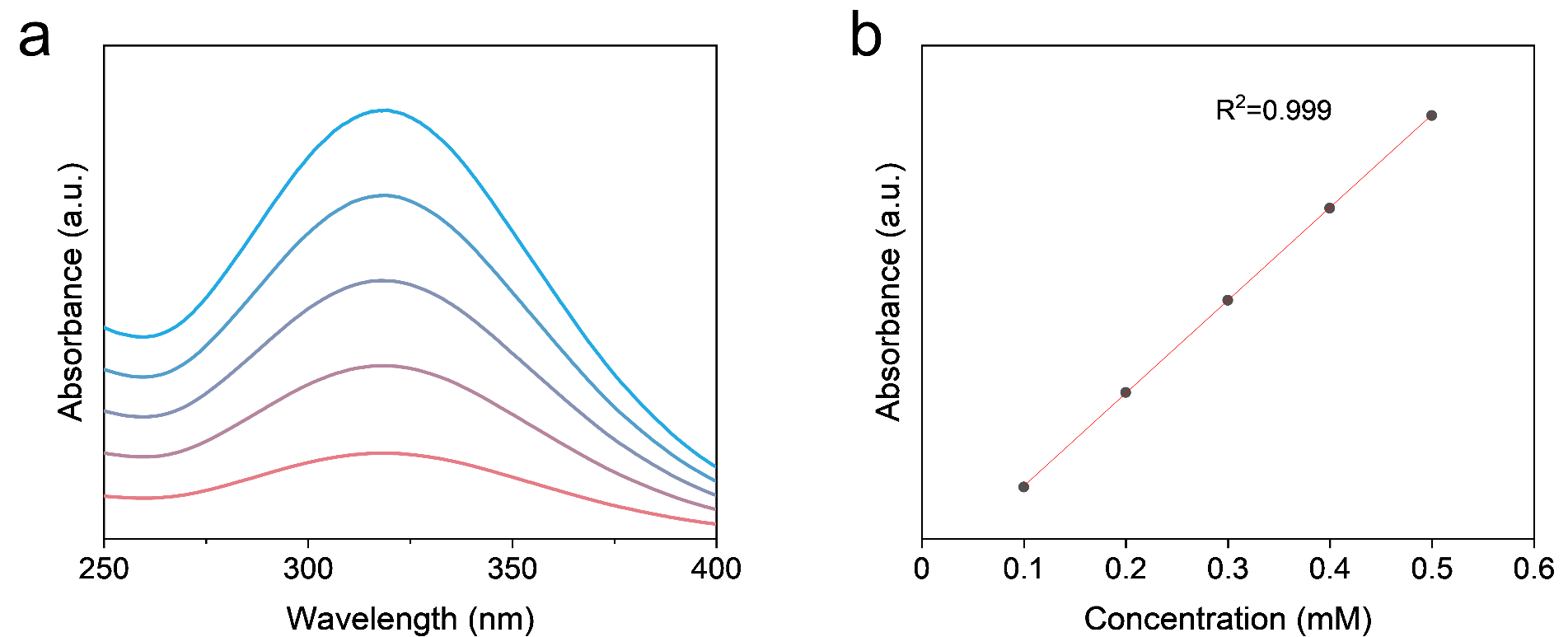


**Figure S32. Standard curves of Ce^4+^ concentration.** (**a**) UV-vis spectra of cerium solutions. (**b**) Corresponding fitted standard curve.


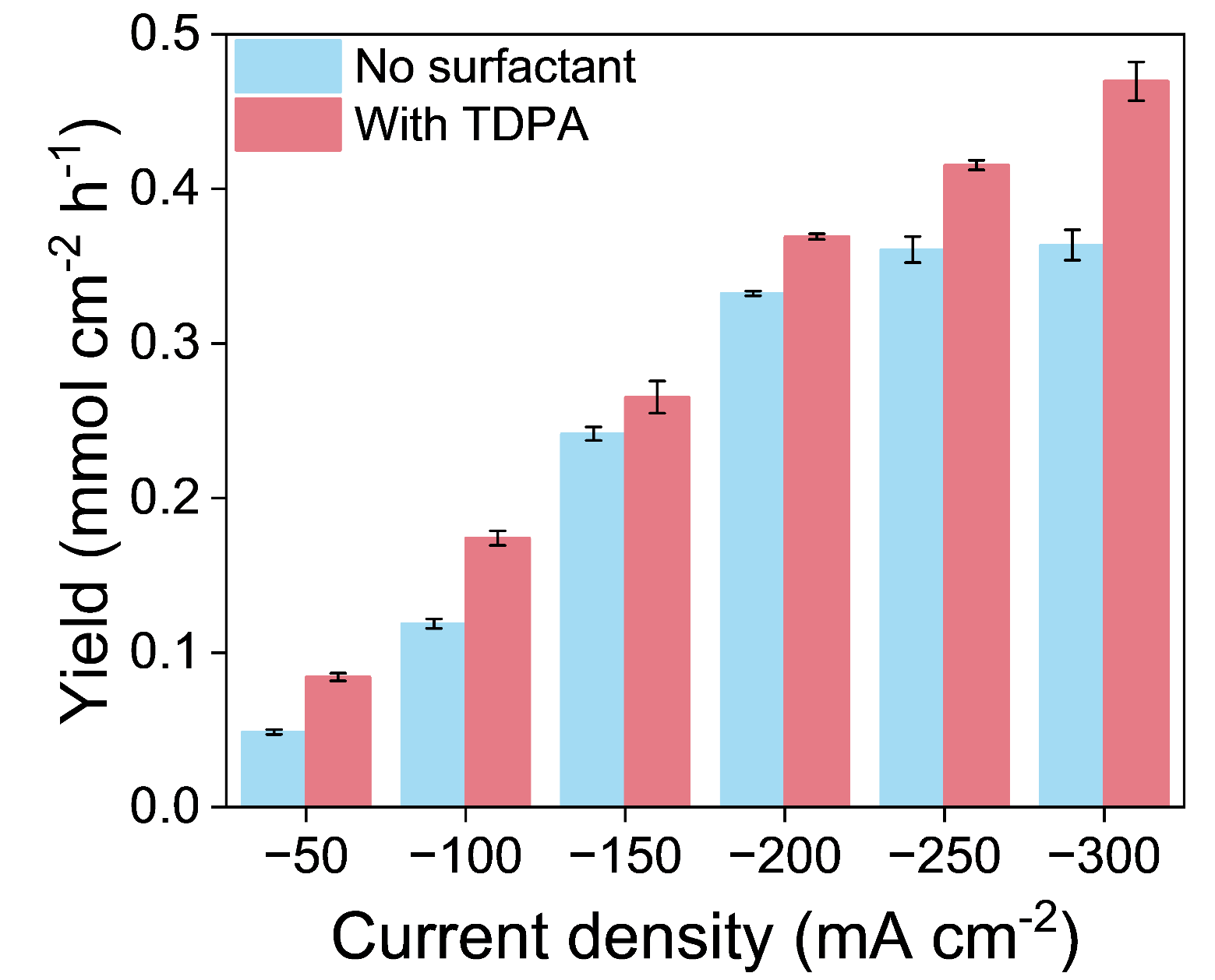


**Figure S33.** The yield of H_2_O_2_ in 0.1 M KOH electrolyte with and without TDPA from −50 to −300 mA cm^−2^.


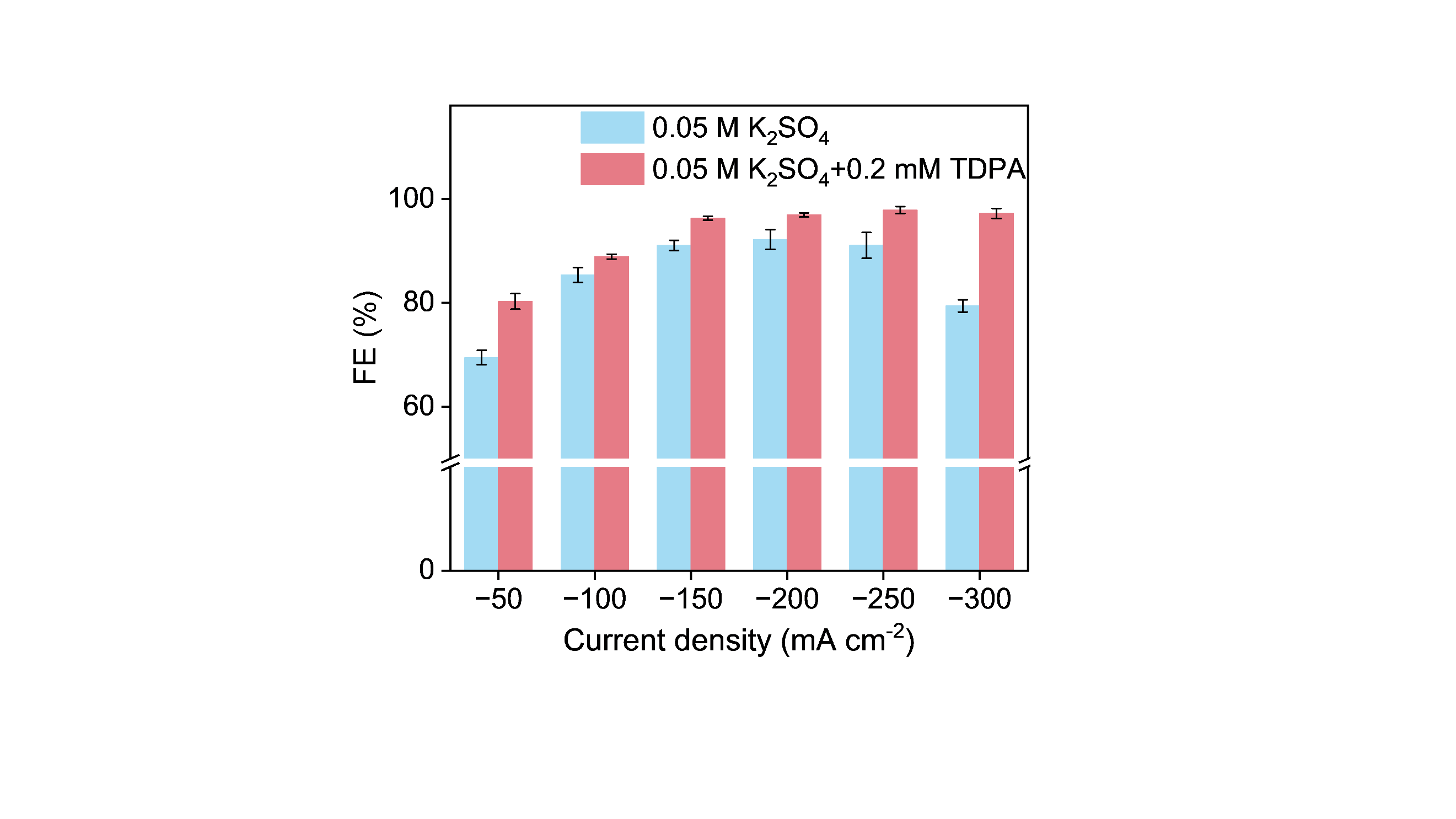


**Figure S34.** Comparison of H_2_O_2_ FE in 0.05 M K_2_SO_4_ electrolytes with and without 0.2 mM TDPA at different current densities.


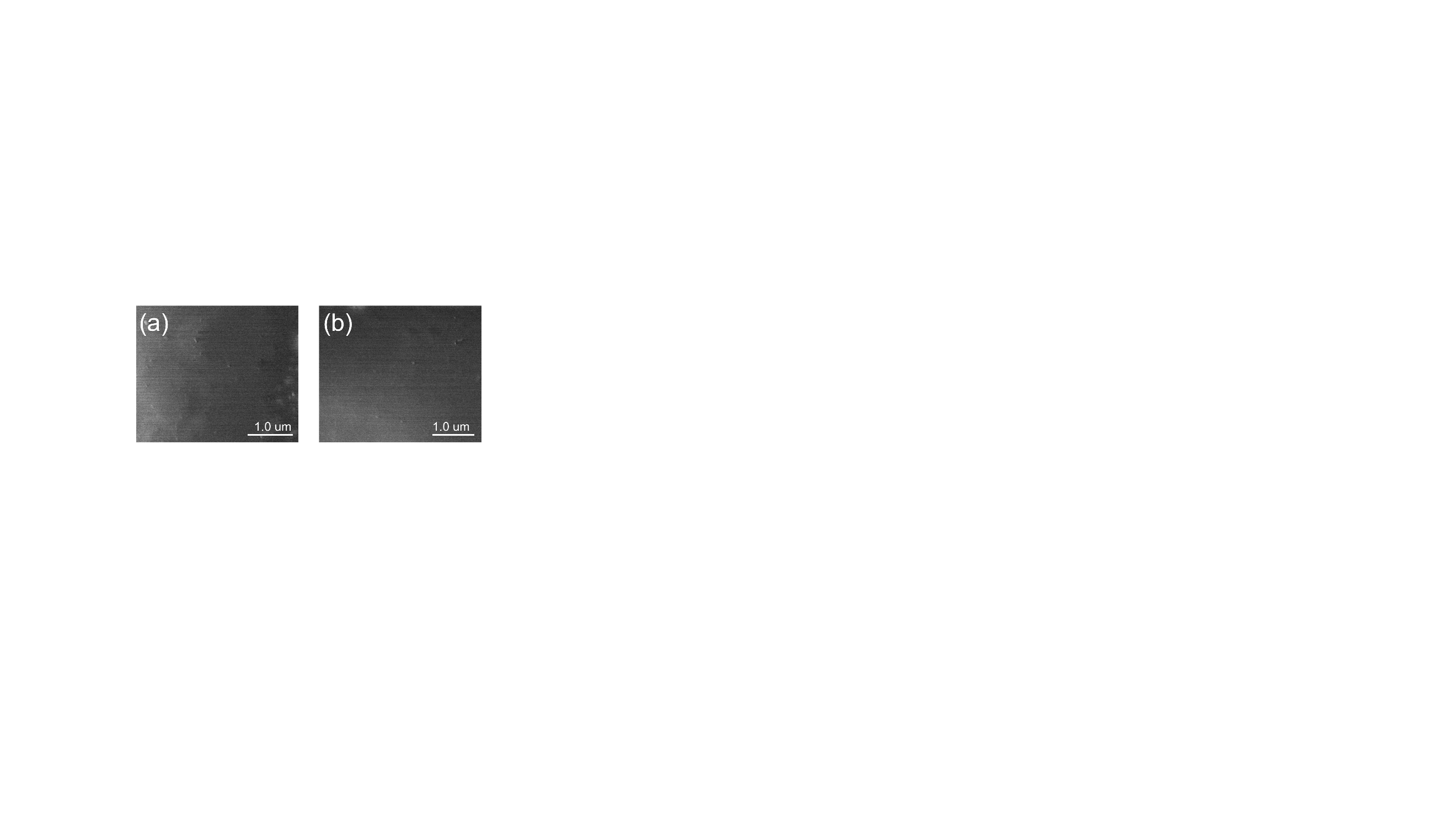


**Figure S35.** SEM image of Nafion 117 membranes (**a**) before and (**b**) after reaction.


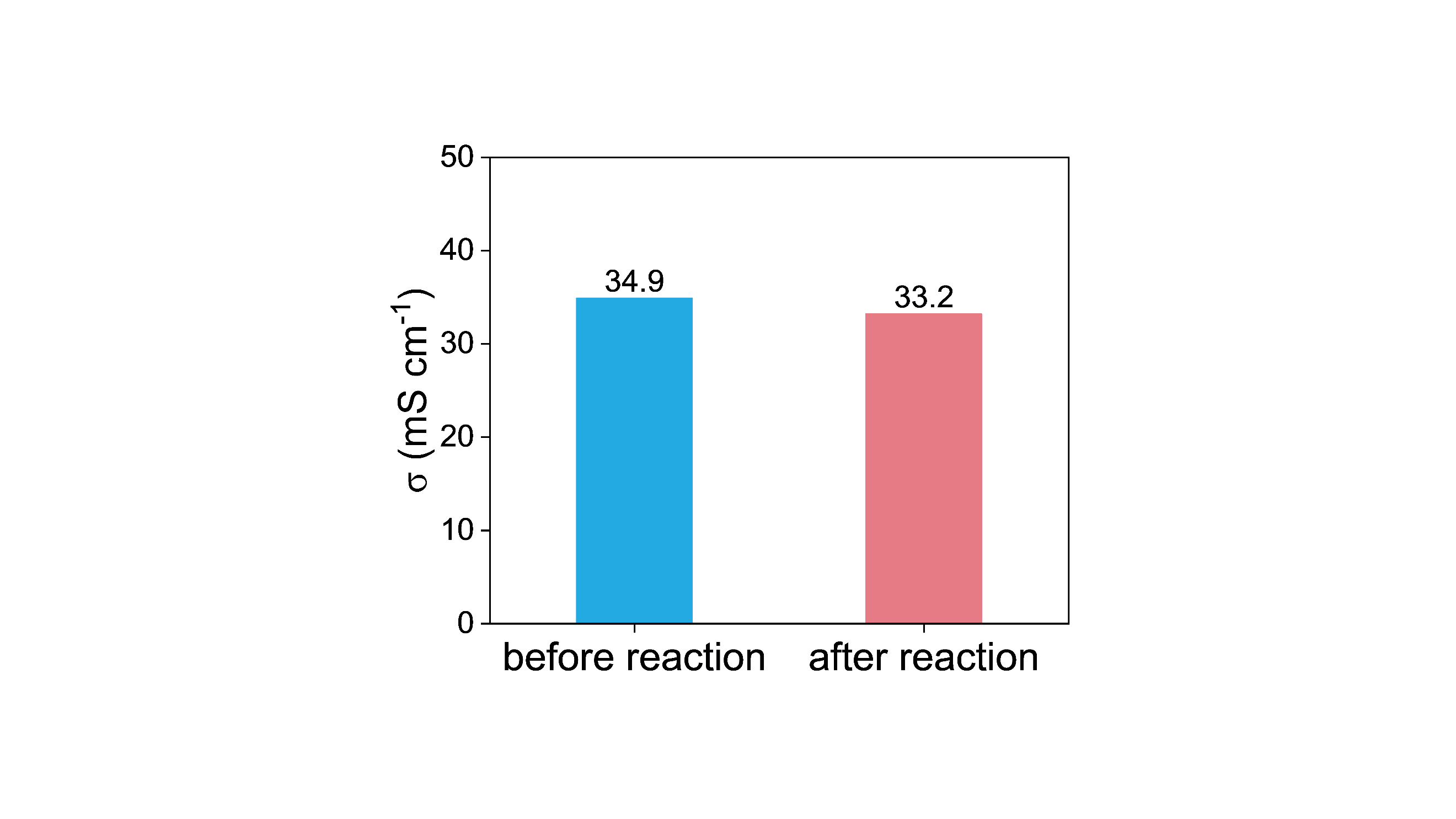


**Figure S36.** Conductivity of Nafion 117 membranes before and after reaction.

**Table S1.** Fitting parameters of EIS in KOH electrolyte at 1600 rpm.

| E (V_RHE_) | R_s_ (Ω) | R_1_ (Ω) | R_2_ (Ω) |
| --- | --- | --- | --- |
| 0.8 | 42.41 | 11238 |  |
| 0.75 | 41.53 | 1056 |  |
| 0.7 | 41.11 | 217.9 |  |
| 0.65 | 41.89 | 84.72 | 73.83 |
| 0.6 | 42.52 | 95.73 | 205.1 |
| 0.5 | 40.55 | 4078 |  |
| 0.4 | 41.01 | 57168 |  |
| 0.3 | 43.43 | 5461 |  |
| 0.2 | 41.36 | 2479 |  |
| 0.1 | 44.11 | 1302 |  |

**Table S2.** Fitting parameters of EIS in KOH electrolyte with TDPA at 1600 rpm.

| E (V_RHE_) | R_s_ (Ω) | R_1_ (Ω) | R_2_ (Ω) |
| --- | --- | --- | --- |
| 0.8 | 41.36 | 29103 |  |
| 0.75 | 42.38 | 3244 |  |
| 0.7 | 43.87 | 561 |  |
| 0.65 | 43.85 | 186.5 | 89.75 |
| 0.6 | 42.57 | 58.69 | 435.5 |
| 0.5 | 41.60 | 10671 |  |
| 0.4 | 41.25 | 10^12^ |  |
| 0.3 | 41.23 | 8738 |  |
| 0.2 | 43.64 | 5671 |  |
| 0.1 | 43.51 | 5577 |  |

**Table S3.** Force field parameters used in this study.

| Bonds | r_0_ (nm) | K (kJ mol^-1^ nm^-2^) |
| --- | --- | --- |
| O–H | 0.096 | 456056 |
| C–C | 0.153 | 186188 |
| C–H | 0.1111 | 258571.2 |
| C–P | 0.189 | 225936 |
| P–O | 0.152 | 334720 |
| Angles | θ_0_ (deg) | K (kJ mol^–1^ rad^–2^) |
| C–C–P | 105.5 | 359.824 |
| H–C–C | 110.1 | 289.5328 |
| O–P–C | 103 | 827.5952 |
| C–C–C | 113.6 | 488.2728 |
| H–O–P | 115 | 251.04 |
| Dihedrals | Phase (deg) | K_d_ (kJ mol^–1^ rad^–2^) |
| C–C–C–P | 180 | 3.93296 |
| P–C–C–H | 0 | 0.04184 |
| C–C–P–O | 0 | 0.2092 |
| H–C–C–H | 0 | 0.92048 |
| C–C–C–C | 0 | 0.395723 |

**Table S4.** Details of simulated models.

|  | 1 M KOH–0 M TDPA | 1 M KOH–0.1 M TDPA |
| --- | --- | --- |
| Number of K^+^ | 40 | 48 |
| Number of OH^–^ | 40 | 40 |
| Number of TDPA | 0 | 4 |
| Number of Water | 2207 | 1935 |
| Equilibrium volume (Å^3^) | 64564.21 | 59174.47 |
| Temperature (K) | 300 | 300 |
| Equilibrium density (kg m^–3^) | 1075.42 | 1094.16 |

**Table S5.** Hydrogen evolution activity.

|  | Free energy  (298.15 K, 0.1 M) | ∆G^sol^ (Hartree) | ∆G^sol^  (kJ mol^-1^) | ∆G^sol^  (kJ mol^-1^) |
| --- | --- | --- | --- | --- |
| H_2_O+H_2_O | −152.936 | 0.590802 | 1551.1496 | 72.0041 |
| H_2_O+OH^−^ | −152.332 |  |  |  |
| TDPA+H_2_O | −1196.2 | 0.618226 | 1623.1537 |  |
| TDPA+OH^−^ | −1195.56 |  |  |  |

# Reference

[1] B. Hess, C. Kutzner, D. van der Spoel, *J. Chem. Theory Comput*. **2008**, *4*, 435–447.

[2] Becke, A. D. Phys. Rev. A Gen. *Phys.* **1988**, *38*, 3098–3100

[3] GRIMME S, EHRLICH S, GOERIGK L. *J. Comput. Chem.* **2011**, *32*, 1456–1465.
